# GeoBioMed perspectives on kidney stone recurrence from the reactive surface area of SWL-derived particles

**DOI:** 10.1038/s41598-022-23331-5

**Published:** 2022-11-01

**Authors:** Lauren G. Todorov, Mayandi Sivaguru, Amy E. Krambeck, Matthew S. Lee, John C. Lieske, Bruce W. Fouke

**Affiliations:** 1 Department of Geology, University of Illinois at Urbana-Champaign, Urbana, IL USA; 2 Carl R. Woese Institute for Genomic Biology, University of Illinois at Urbana-Champaign, Urbana, IL USA; 3 Cytometry and Microscopy to Omics Facility, Roy J. Carver Biotechnology Center, University of Illinois at Urbana-Champaign, Urbana, IL USA; 4 Department of Urology, Mayo Clinic, Rochester, MN USA; 5 Department of Urology, Northwestern University Feinberg School of Medicine, Chicago, IL USA; 6 Division of Nephrology and Hypertension, Mayo Clinic, Rochester, MN USA; 7 Department of Laboratory Medicine and Pathology, Mayo Clinic, Rochester, MN USA; 8 Department of Biomedical and Translational Sciences, Carle Illinois College of Medicine, University of Illinois at Urbana-Champaign, Urbana, IL USA; 9 Roy J. Carver Biotechnology Center, University of Illinois at Urbana-Champaign, Urbana, IL USA; 10 Department of Evolution, Ecology and Behavior, University of Illinois at Urbana-Champaign, Urbana, IL USA

**Keywords:** Urology, Urological manifestations

## Abstract

Shock wave lithotripsy (SWL) is an effective and commonly applied clinical treatment for human kidney stones. Yet the success of SWL is counterbalanced by the risk of retained fragments causing recurrent stone formation, which may require retreatment. This study has applied GeoBioMed experimental and analytical approaches to determine the size frequency distribution, fracture patterns, and reactive surface area of SWL-derived particles within the context of their original crystal growth structure (*crystalline architecture*) as revealed by confocal autofluorescence (CAF) and super-resolution autofluorescence (SRAF) microscopy. Multiple calcium oxalate (CaOx) stones were removed from a Mayo Clinic patient using standard percutaneous nephrolithotomy (PCNL) and shock pulse lithotripsy (SPL). This produced approximately 4–12 mm-diameter PCNL-derived fragments that were experimentally treated ex vivo with SWL to form hundreds of smaller particles. Fractures propagated through the crystalline architecture of PCNL-derived fragments in a variety of geometric orientations to form rectangular, pointed, concentrically spalled, and irregular SWL-derived particles. Size frequency distributions ranged from fine silt (4–8 μm) to very fine pebbles (2–4 mm), according to the Wentworth grain size scale, with a mean size of fine sand (125–250 μm). Importantly, these SWL-derived particles are smaller than the 3–4 mm-diameter detection limit of clinical computed tomography (CT) techniques and can be retained on internal kidney membrane surfaces. This creates clinically undetectable crystallization seed points with extremely high reactive surface areas, which dramatically enhance the multiple events of crystallization and dissolution (*diagenetic phase transitions*) that may lead to the high rates of CaOx kidney stone recurrence after SWL treatment.

## Introduction

The worldwide prevalence and incidence of kidney stones continues to increase, which can range from asymptomatic incidental findings of limited concern to painful recurrent disorders and even chronic kidney disease^1^. This is observed in all cohorts of sex, age, ethnicity, and race, while exhibiting substantial geographic variability that varies from 3 to 15% in the United States to 1–19% in Asia, 4% in South America, 5–10% in Europe, and 20–25% in the Middle East^1–14^. A significant proportion of the identification of these global increases in kidney stone incidence result from improved medical imaging using non-contrast computed tomography (NCCT)^15–20^. In addition, the accurate diagnosis of kidney stone disease is further complicated by uncertainties in diagnostic codes, self-reporting, stone terminology and classification, risk factor identification, and other uncertainties associated with the prediction and monitoring of stone recurrence^1^. A definitive diagnosis of symptomatic kidney stones therefore still fundamentally depends upon an actual stone being imaged, observed when surgically removed, or after being voided^1^.

As a result of these complex intertwined factors, shock wave lithotripsy (SWL) remains a common non-invasive clinical intervention for kidney stones globally, because it is relatively low-cost and approximately 60% effective in fragmenting and at least partially eliminating stones less than approximately 11–20 mm in diameter^9,16,21–24^. Despite clinical CT techniques that currently approach 1 mm resolution, patients with SWL-derived residual stone particles that are ≤ 3–4 mm in diameter are considered to be “clinically insignificant residual fragments” because they are assumed to be able to spontaneously pass. Therefore, patients with fragments up to this size are classified as being in a “stone free state” and considered to not require further treatment^15–18,25^. In contrast, post-SWL treatment patients also commonly experience high stone recurrence rates that reach ~ 78%^26,27^, which may at least partially result from crystallization at sites of SWL-induced tissue damage as observed in renal histology section^28^. Post-SWL treatment recurrence has also been postulated, but not yet experimentally proven, to be caused by crystal regrowth from residual SWL-derived particles that have been found throughout the anatomical structure of the kidney^9,17,18,21,22,29–37^.

**Figure 1:**
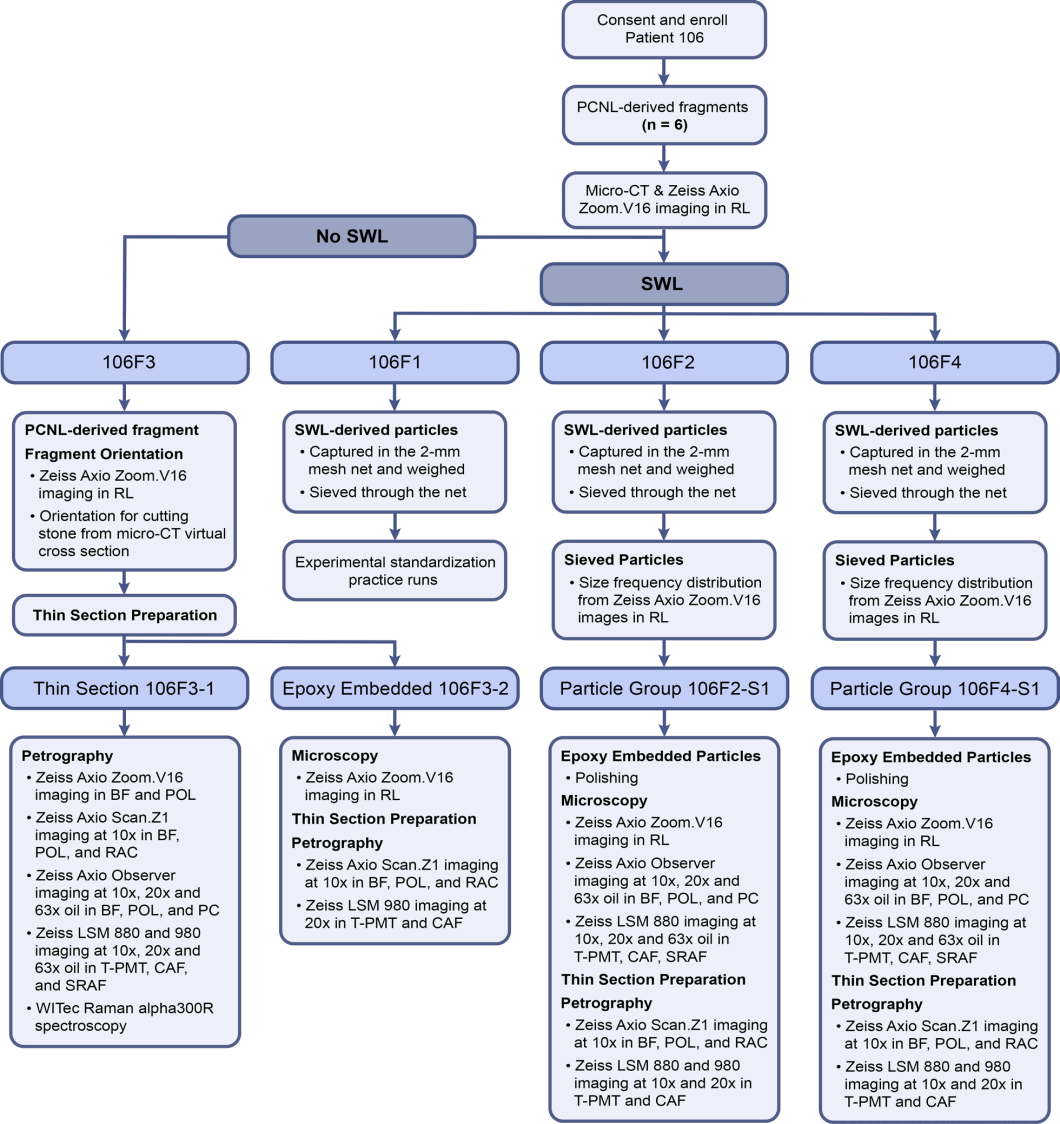
Flow chart of experimental study (see explanation in Supplementary Materials and Methods). Acronyms include: *SWL* shock wave lithotripsy, *PCNL* percutaneous nephrolithotomy, *CT*, computed tomography, *RL* reflected light, *BF* brightfield, *POL* polarization, *RAC* ring aperture contrast, *PC* phase contrast, *T-PMT* transmitted light photomultiplier tube, *CAF* confocal autofluorescence, *SRAF* super-resolution autofluorescence, and *×10, ×20, ×63* microscope objective magnification.

The present study was undertaken to experimentally quantify SWL-induced kidney stone fragmentation from the perspective of an integrative geological, biological, and medical approach called *GeoBioMed*^38–42^. Of direct relevance for better understanding post-SWL recurrence, GeoBioMed incorporates practical and theoretical geoscience approaches that characterize grain size and predict resulting chemical reactivity^43–45^. Six calcium oxalate (CaOx) stone fragments were collected from a patient undergoing standard percutaneous nephrolithotomy (PCNL) with shock pulse lithotripsy (SPL) (Fig. 1), which is typically applied to stones greater than approximately 11 mm in diameter^16^. These *PCNL-derived fragments* were further broken-down ex vivo on a Dornier Delta^®^ III lithotripter to produce a wide array of smaller *SWL-derived particles*. Weight loss during SWL experimentation was determined (defined as [PCNL-derived fragment weight] − [total SWL-derived particle weight]) and size frequency distributions were measured from reflected light images of loose SWL-derived particles^46,47^. A subset of these SWL-derived particles was embedded in epoxy plugs and the polished surface analyzed using: (1) 250 nm-resolution microscopy (reflected light—RL; brightfield—BF; ring aperture contrast—RAC; phase contrast—PC; polarization—POL; transmitted light photomultiplier tube—T-PMT; confocal autofluorescence—CAF); and (2) 140 nm-resolution microscopy (T-PMT; super-resolution auto-fluorescence—SRAF). Each epoxy plug was made into 25 μm-thick doubly polished uncovered thin sections for 140 nm-resolution microscopy (BF, POL, RAC, T-PMT, CAF). Mineralogy was determined with a combination of this high- and super-resolution microscopy and Raman spectroscopy. Results indicate that: (1) the original crystal growth structure and post-formational crystallization and dissolution alteration (*diagenetic phase transitions*) combine to create the crystalline architecture of CaOx kidney stones; (2) this provides an essential contextual framework within which to identify and track how SWL fracture patterns develop^42^; and (3) small particles (< 3–4 mm-diameter) undetectable with current clinical CT and ultra sound techniques are produced by SWL treatment, which can be systematically categorized using the geological Wentworth grain size scale^48^. Results suggest that the dramatic increase in reactive surface area created by these small SWL-derived particles, which become distributed throughout the kidney, dramatically enhance the multiple events of diagenetic phase transitions that result in high rates of stone recurrence after SWL treatment.

## Results and discussion

### Crystalline architecture of PCNL-derived fragments

**Figure 2:**
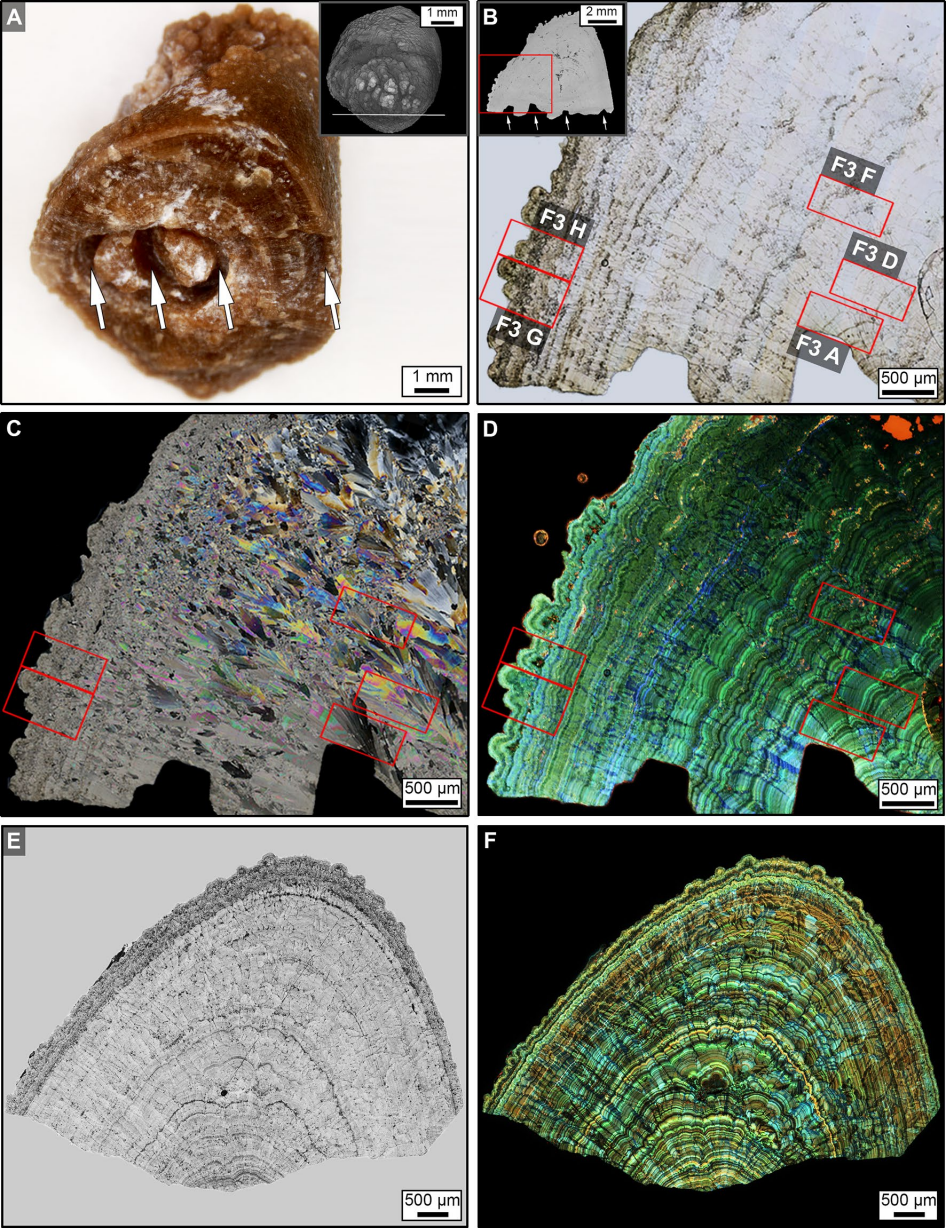
Three-dimensional (3D) external morphology of kidney stone fragment 106F3 and two-dimensional (2D) internal crystalline architecture of thin section 106F3-1 and 106F3-2. (**A**) 3D reflected light (RL) image of the entire stone showing notches (white arrows) resulting during PCNL SPL procedure. Weight data presented in Supplementary Table S1. Inset is a 3D computed tomography (CT) external surface rendering of entire stone from a different orientation showing the line of section (white line). Thin section 106F3-2 was prepared ~ 500 μm below the line of section. (**B**) Brightfield (BF) image of 2D virtual thin section 106F3-1 made at position shown in the line of section show in the inset of (**A**), exhibiting a complete history of earliest-to-latest stone growth crystallization. Red boxes indicate the locations of enlargements shown in Fig. 3. Inset is a 2D virtual CT cross-section taken through line of section shown in (**A**). White arrows show PCNL and SPL notches that correspond to (**A**), and red box indicated enlargement in (**B**). (**C**) Polarization (POL) image of (**B**). Red boxes indicate the location of enlargements shown in Fig. 3. (**D**) Tiled confocal autofluorescence (CAF) image of merged pseudo-colored red, green, and blue (RGB) channels indicate that the COM cortex (COM_C_) is composed of nano-layering. Red boxes indicate the location of enlargements shown in Fig. 3. (**E**) T-PMT of thin section 106F3-2 exhibiting the original internal crystalline architecture of the PCNL-derived 106F3 stone fragment. (**F**) Tiled CAF image of merged pseudo-colored RGB channels of (**E**).

**Figure 3:**
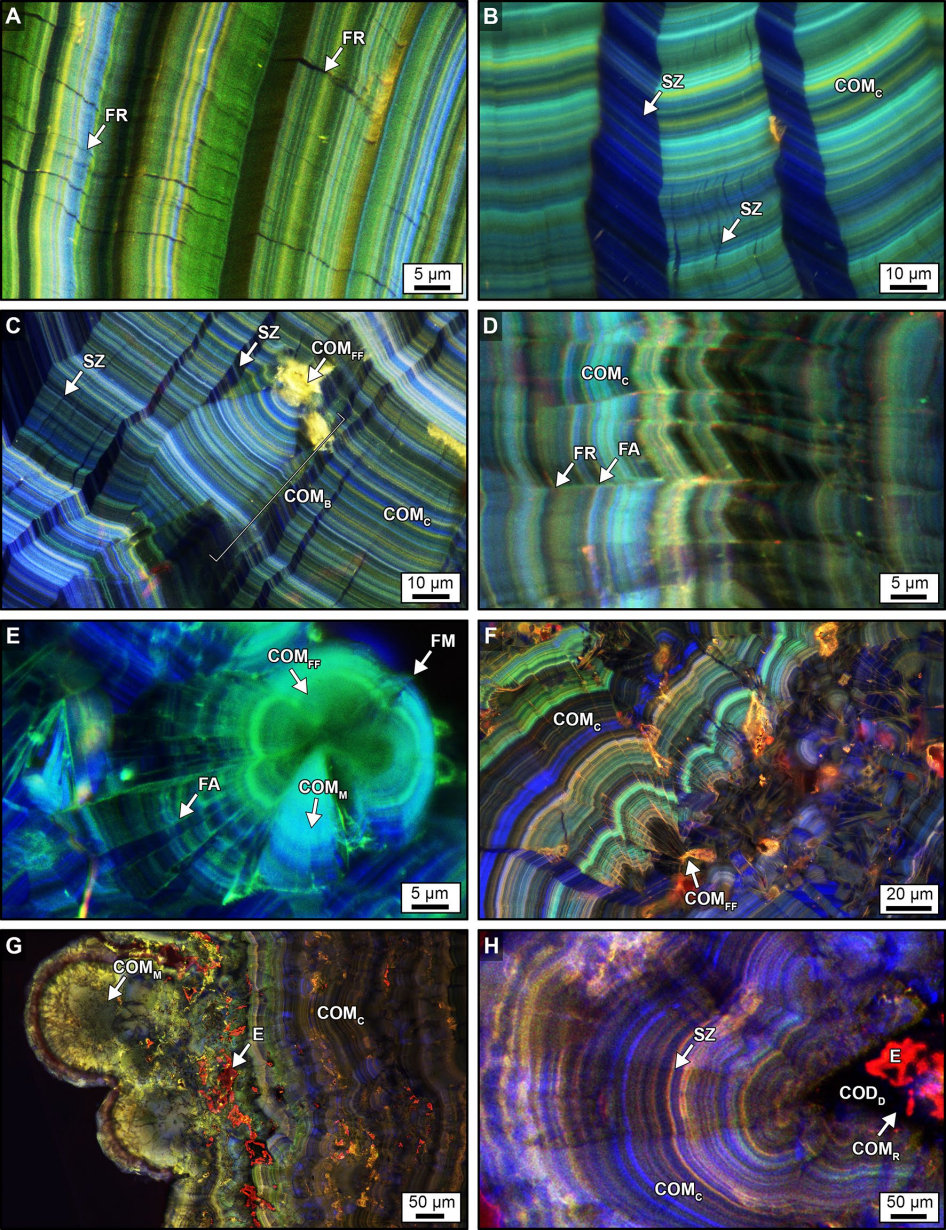
Original crystalline architecture of calcium oxalate (CaOx) PCNL-derived fragments. (**A,D,E**) SRAF images. (**B,C,F,G,H**) CAF images. Labels indicate: *FR* fracture, *FA* fault *L* lath, *SZ* sector zone, *COM*_*FF*_ free-floating COM, *COM*_*C*_ COM cortex, *COM*_*B*_ bundles of COM radiating from a COM_FF_, *COM*_*M*_ mimetic replacement COM, *COM*_*R*_ replacement COM, *COD*_*D*_ dissolved COD; and E, red AF embedding epoxy. Image locations shown in Figs. 2B–D and 4C and corresponding T-PMT images presented in Supplementary Fig. S3.

The high- and super-resolution optical, laser, X-ray microscopy, and Raman spectroscopy conducted in the present study indicate that the original PCNL-derived fragments have a crystalline architecture that is consistent with those previously observed in CaOx kidney stones^38–42^. Following the approaches presented in these previous studies, the term “crystalline architecture” is herein used to refer to stone structure (crystal size, shape, intergrown morphologies), stratigraphy (crystal and organic matter layering), diagenetic phase transitions (post-depositional dissolution and recrystallization), and paragenesis (historical sequence of formational events). Therefore, the approaches and terminology of GeoBioMed^38–42^ have been directly adopted and applied in the present study. RL microscopy and X-ray computed tomography (CT) indicate that the original PCNL-derived fragments are irregular 4–12 mm-diameter crystalline aggregates that exhibit SPL probe impressions and breakage patterns (Figs. 2A,B, SFigs. 1, 2). Each PCNL-derived and SPL-derived fragment in the present study is primarily a high-density calcium oxalate monohydrate (COM; Whewellite; CaC_2_O_4_·H_2_O) cortex (COM_C_) composed of high-frequency alternations of organic-matter-rich nanolayers (peptides, proteins, and other cellular molecules) and COM mineral-rich nanolayers (Fig. 2B–F)^49–52^. All six of the PCNL-derived fragments were impacted to varying degrees by ultrasonic wave energy and intermittent shockwaves from use of an SPL probe. This includes breakage surfaces and notch formation on the exterior of some PCNL-derived fragments (Fig. 2A,B, SFigs. 1, 2), as well as rare occurrences of fine fracturing within COM_C_ that are exclusively observed adjacent to the SPL notches (Fig. 3A).

Individual crystal faces (*sector zones*) within COM_C_ are common and form as the result of disequilibrium precipitation, during which ions and organic matter are differentially incorporated on age-equivalent crystal growth faces (Fig. 3B,C,H)^38–42,53^. Free-floating COM crystals (COM_FF_) are entombed, either individually or in clusters, on growing COM_C_ concentric surfaces that seed the growth of *radiating crystal bundles* (COM_B_) that either truncate or redirect the growth of sector zones (Fig. 3C,F). Additionally, COM_C_ commonly exhibits repeated in vivo events of crystal fracturing (cracking) and faulting (displacement across the fractures) to form laths with discontinuous layering (Fig. 3D,E). COM_C_ also exhibits Ångstrom-scale dissolution and recrystallization (*mimetic replacement*, COM_M_) (Fig. 3E,G). The outermost margins of the PCNL-derived fragments are composed of calcium oxalate dihydrate (COD; Weddellite; CaC_2_O_4_·2H_2_O) crystals that were encrusted by COM_C_, experienced COD dissolution (COD_D_), and were partially filled with replacement COM (COM_R_) (Fig. 3H).

### Fracture geometries of SWL-derived particles

The crystalline architecture of the SWL-derived particles, as revealed by CAF and SRAF microscopy, provides a high-fidelity crystalline framework within which to characterize SWL-induced fracture geometries (Figs. 5, 6). Importantly, previous studies using scanning electron microscopy (SEM) on SWL-derived particles provided valuable information on external and internal CaOx crystal growth and fracturing morphologies^49^. However, the SEM tool is inherently limited compared to the detailed information on crystalline architecture that is provided by the CAF and SRAF microscopy^38–42^. SWL-induced fractures were observed in the present study to propagate in a variety of perpendicular, oblique, and concentric-parallel trajectories relative to the nanolayered crystalline architecture of the original PCNL-derived fragments (Figs. 5, 6; SFigs. 6–10). These observations have been integrated to establish a systematic nomenclature of fracture morphologies for SWL-derived particles that include: (1) *rectangular particles* with fracture margins formed perpendicular to the COM_C_ concentric nanolayer stratigraphy (Fig. 5A), parallel to radiating sector zones (Fig. 6B,D), and cross-cutting COM mimetic replacement (COM_M_) crystals (Fig. 6G)^49,54–56^; (2) *pointed particles* that form from fractures perpendicular, oblique, and parallel to the COM_C_ concentric nanolayer stratigraphy (Fig. 5B–H), which merge at angles of 60°–120° to form arrowhead-like tips (Figs. 5B–H, 6A,D) that are similar in appearance to Hertzian-cone conchoidal fractures caused by surface radial tensile stress in silicates and metals^56–58^; (3) *concentrically spalled particles* created by inter- and/or intracrystalline fracturing along concentric organic matter-rich COM_C_ nanolayers via cohesive-zone brittle microfracture spallation (Fig. 5F,G)^35,54,55^; and (4) *irregular surface particles* created by coalescing microfractures that cause irreversible fatigue damage and little plastic deformation during the application of cyclic tensile stress^54–56,59,60^ at the margins of original COM crystal bundles that initially grow from COM free floating (COM_FF_) crystals after landing on growing COM_C_ surfaces (Fig. 6B–H). These CAF- and SRAF-defined fracture categories enhance and improve upon observations completed in earlier studies exclusively using SEM^49^.

### SWL-derived particle size distributions

During the course of the 72-h H_2_O porosity saturation of the original PCNL-derived fragments^61^, the weight of both 106F2 and 106F4 increased approximately 10% (Fig. 7A). The larger PCNL-derived fragment 106F2 was exposed to six sequential ex vivo 100-shock SWL treatments with a consistent Level 3 intensity at a rate of 90 shocks per minute (Fig. 7). The smaller PCNL-derived fragment 106F4 was subjected to only two 100-shock treatments at the same shockwave intensity and rate (Fig. 7). The SWL-derived particles produced by each 100-shock treatment were sieved with the 2 mm-mesh net in the calibration container of the lithotripter, weighed, and imaged (SFig. 3, STable 1). The weight percent of SWL-derived particles captured in the 2 mm-mesh net was observed to decrease from approximately 50–85% after the first 100-shock treatment and eventually reach a 0% decrease after progressive treatments. This is consistent with previous observations of weight loss with increasing shock wave treatments^50^. The SWL-derived particle fractions small enough to pass through the 2 mm-mesh net were trapped on a 0.47 μm filter, imaged, quantified, and classified according to the Wentworth grain size scale (Fig. 7B,C)^48,62,63^. These analyses indicate that the SWL-derived particles from both 106F2 and 106F4 that passed through the 2 mm-mesh net are in size classes that range from very fine silt (4.998 μm) to very fine pebbles (2.926 mm) (Fig. 7B,C, STable 2). The SWL-derived particles from both 106F2 and 106F4 exhibit right-skewed (positive) normal size frequency distributions (after Garcia, 2008) with size class modes of fine sand (Fig. 7B,C)^64^. While small clay-sized (< 4 µm-diameter) SWL-derived particles were not detected in the present study despite the use of 0.47 µm filters (Fig. 7), it is possible that some unknown amount of this fine grain size class may have been lost while decanting between sequential stages of SWL experimentation.

### Implications

Experimental results in the present study indicate that each SWL treatment of CaOx PCNL-derived fragments produced 5 µm- to 2 mm-diameter SWL-derived particles that range on the Wentworth grain size scale from very fine silts through sands and very fine pebbles (mode = 125–250 µm-diameter fine sands; Fig. 7). The majority of these SWL-derived particles are significantly below the 3–4 mm-diameter detection limit of clinical non-contrast computer tomography scans^20,65,66^. As discussed here, results from the present study, as well as inference made from previous studies, indicate that these small SWL-derived particles are likely to increase the chance for post-SWL treatment stone recurrence^67–70^. Therefore, the common clinical practice of using negative computed tomography screens to identify and declare patients as “kidney stone free” with “clinically insignificant residual fragments”^17,18,24,29,69,71–76^, should be fundamentally reevaluated.

In natural environmental waters, the primary factors controlling the rate and extent of mineral precipitation and dissolution reactions include mineralogical stability, fluid saturation state, and the amount of surface area available per unit mass of mineral grains^43–45,77^. Furthermore, fine crystalline structure (*crystalline architecture*) and crystal aggregate grain size play influential roles in both the primary (*original*) crystallization and secondary physical, chemical, and biological alteration (*diagenesis*)^42,78,79^ of the kidney stone deposits. The increase in total surface area per gram, as both grain size and volume decrease, can be approximated as (Walter and Morse)^45^:

1 $$\mathrm{A }= \frac{\lambda }{\rho *V}=\left(\frac{\beta }{\rho }\right){r}^{-1},$$

where A = specific surface area (SSA_p_) of each particle per unit mass (g), $\lambda$ = particle surface area (also known as A_p_) per unit mass (g), which is approximated as a sphere using a spherical radius (r) derived from grain size: A_p_ = $4\pi {r}^{2}$, V = volume of each particle (also known as V_p_) as a function of equivalent spherical radius (r): V_p_ = $\frac{4}{3}\pi {r}^{3}$, $\rho$ = bulk density of material (COM)^80^ = 2.12 g/cm^3^, $\beta$ = shape factor for sphere or cube, which is 3 based on surface to volume ratios and assumes geometrically equidimensional particles.

**Figure 4:**
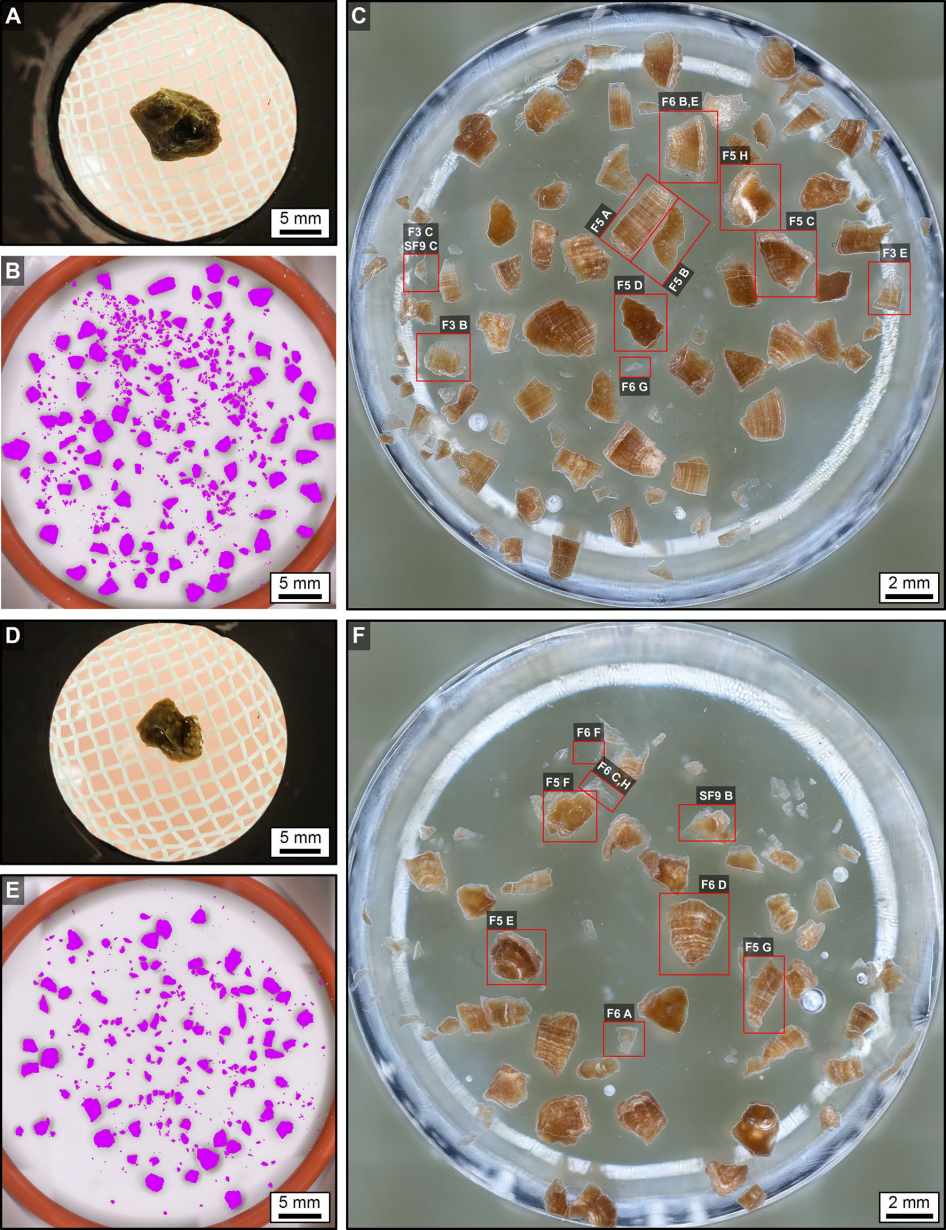
PCNL-derived fragments 106F2 (**A**) and 106F4 (**D**) that underwent SWL to produce particle groups 106F2-S1 (**B**,**C**) and 106F4-S1 (**E**,**F**). Particles derived from the first 100 SWL shocks were imaged (**B**,**E**) and embedded in an epoxy plug (**C**,**F**). (**A**,**D**) RL image of PCNL-derived fragment suspended within a 2 mm-mesh net basket prior to SWL treatment. Corresponding figures of 3D external stone morphology, 2D internal crystalline structure, and RL images during experimentation presented in Supplementary Figs. S2 and S4. (**B**,**E**) RL image of loose SWL-derived particles that were hand-traced and artificially colored pink for precise pixel selection and grain size quantification. (**C**,**F**) RF image of SWL-derived particles embedded in epoxy and polished prior to thin section preparation (red boxes indicate locations of enlargements shown in Figs. 3, 5, 6, Supplementary Fig. S9).

**Figure 5:**
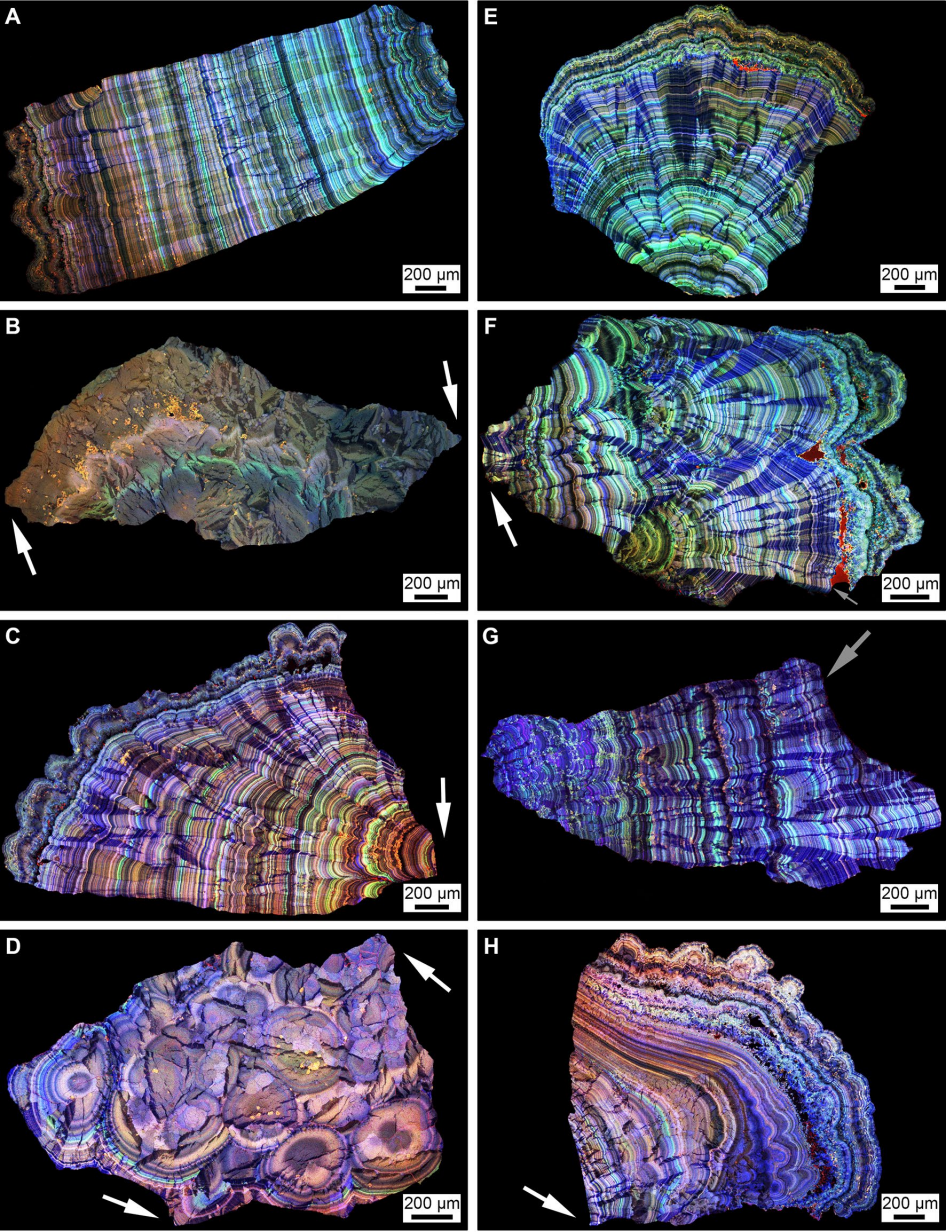
Geometries of SWL-derived particles from groups 106F2-S1 and 106F4-S1 (Fig. 4). These detailed crystal growth structures and fracture patterns, observed in thin section, are analogous to those observed on the polished epoxy plugs (Figs. 3, 6). Red AF emitted from embedding epoxy has been removed from around, but not within each fragment, and replaced with a black background. (**A**–**H**) CAF images of merged pseudo-colored RGB channels showing SWL fracture geometries that crosscut the original CaOx crystalline architecture. SWL shock fractures propagate at perpendicular and oblique angles with respect to the original crystalline architecture, often converging to form angles of 60°–120° ((**B**,**C**,**D**,**F**,**H**), white arrows). Spalling along concentric crystalline layering is also observed ((**F**,**G**), grey arrows). SWL-derived particle locations shown in Fig. 4 and Supplementary Fig. S5 and corresponding BF and POL figures presented in Supplementary Figs. S6 and S7.

**Figure 6:**
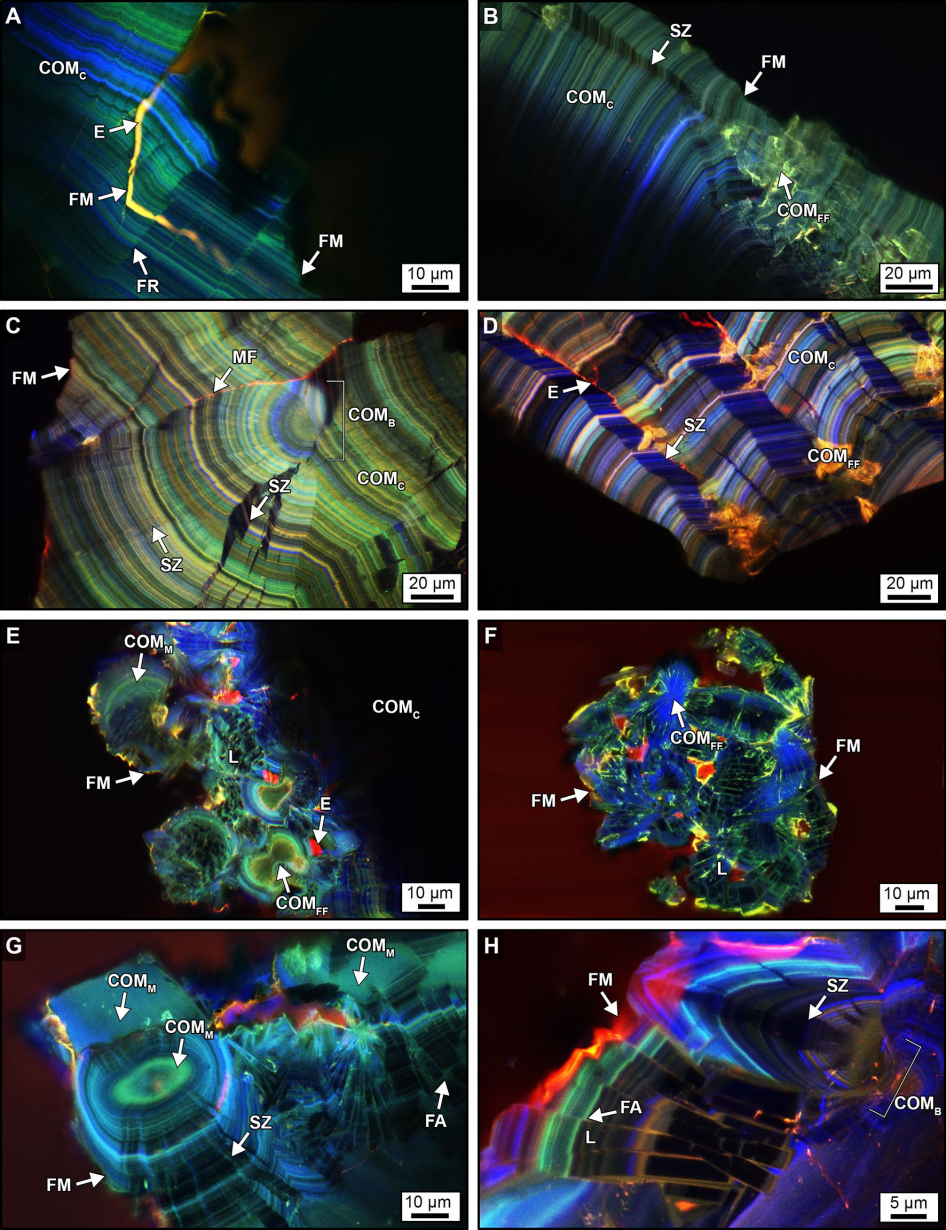
Crystalline architecture and fracture patterns of SWL-derived particles from groups 106F2-S1 and 106F4-S1 embedded in the epoxy plug. (**A**–**G**) CAF images. (**H**) SRAF image. Labels indicate: *FR* fracture, *FM* fracture margin, *FA* fault *L* lath, *SZ* sector zone, *COM*_*FF*_ free-floating COM, *COM*_*C*_ COM cortex, *COM*_*B*_ bundles of COM radiating from a COM_FF_, *MF* microfractures, *COM*_*M*_, mimetic replacement COM; and E, red AF embedding epoxy. Image locations shown in Fig. 4 and corresponding T-PMT figure presented in Supplementary Fig. S8.

**Figure 7:**
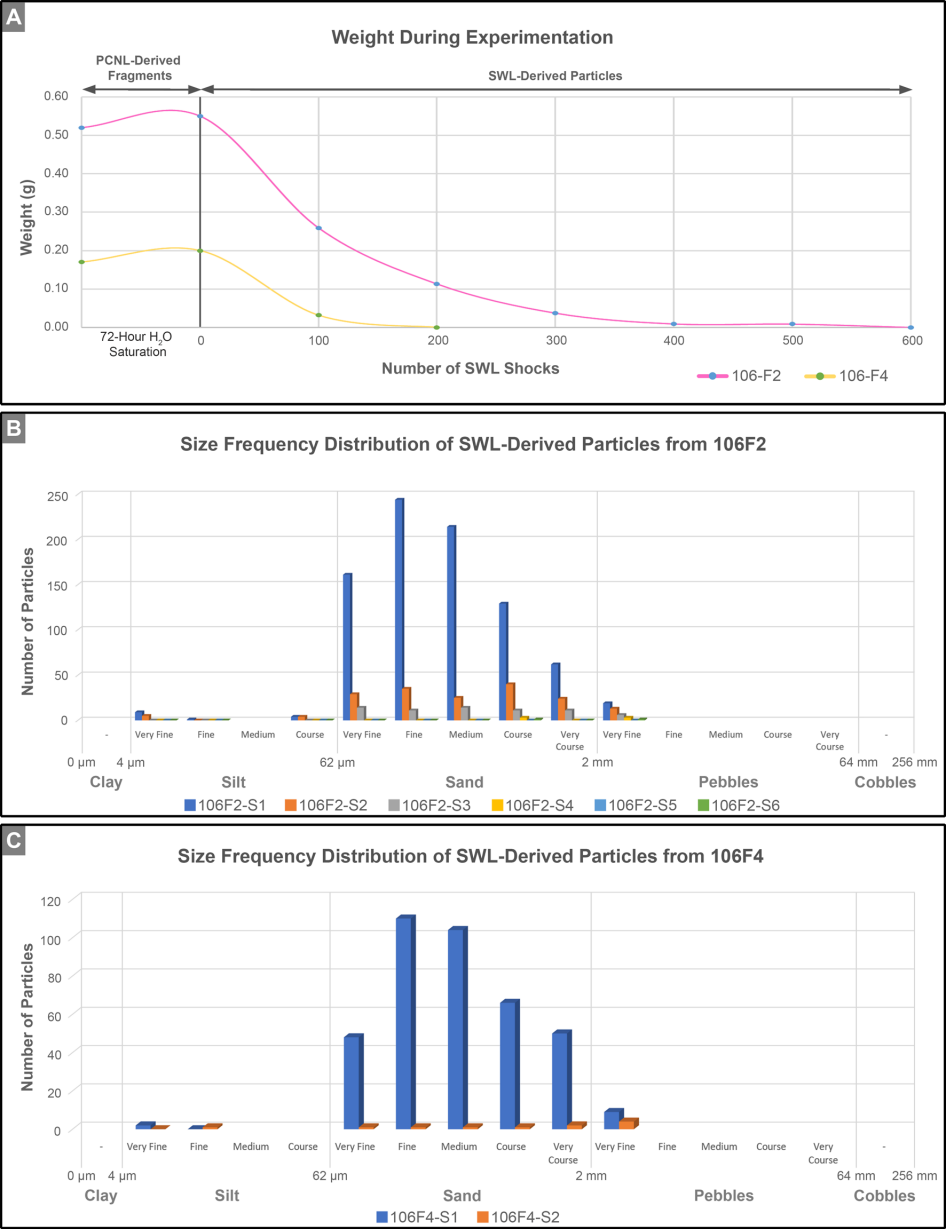
Weight changes and size frequency distributions of SWL-derived particle groups 106F2 and 106F4. (**A**) Change in weight of PCNL-derived fragments 106F2 and 106F4 after 72-h of H_2_O saturation prior to SWL experimentation, and weight changes during experimentation in SWL-derived particles during each incremental 100-shock treatment. Corresponding RL images and data are presented in Supplementary SFig. 4 and Supplementary STable 1. (**B**) Wentworth grain size frequency distribution of SWL-derived particles from 106F2 after six 100-shock treatments. (**C**) Wentworth grain size frequency distribution of SWL-derived particles from 106F4 after two 100-shock treatments. Corresponding data presented in Supplementary STable 2.

**Figure 8:**
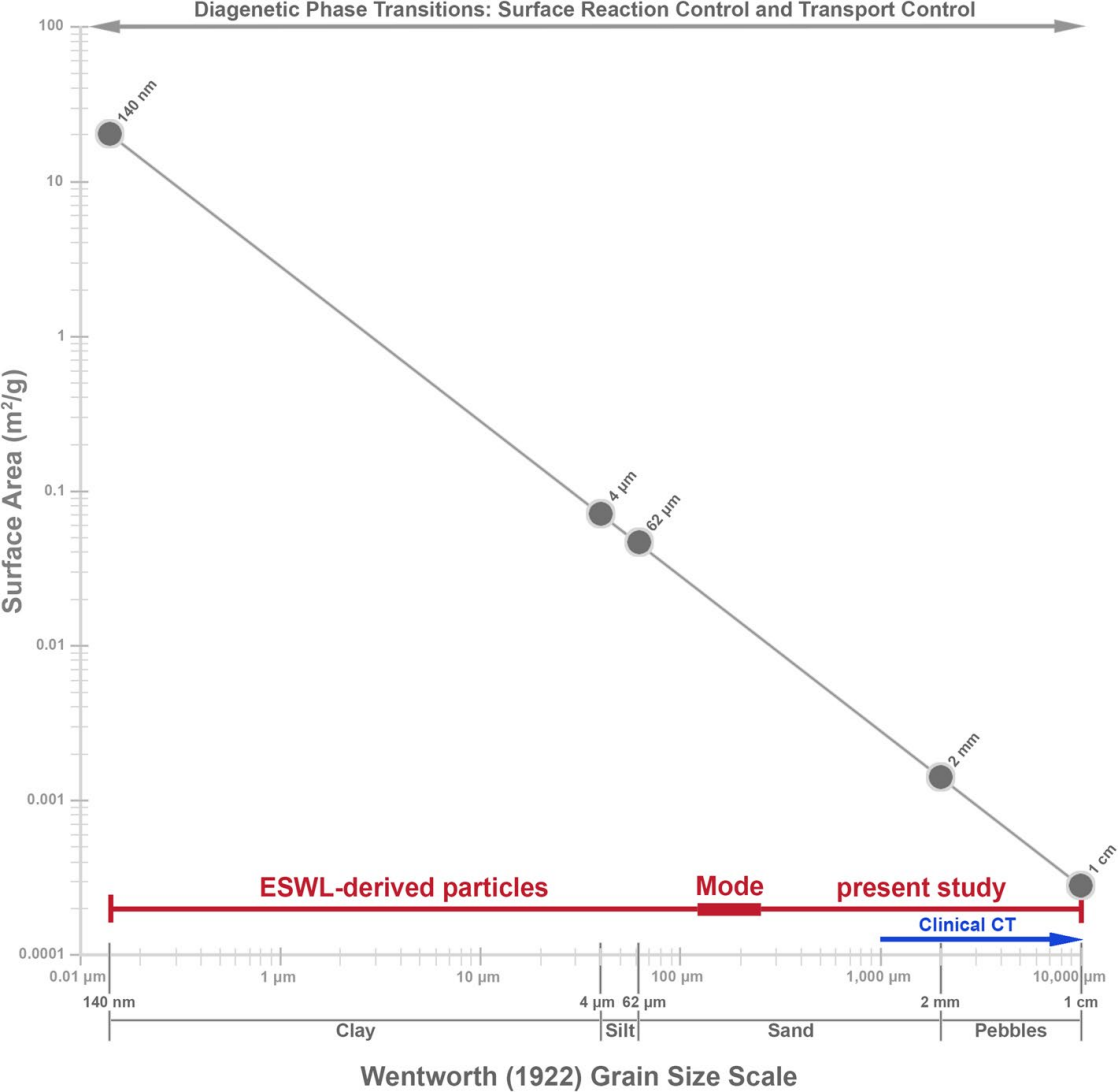
Log plot of specific surface area (m^2^/g) as a function of grain diameter (in microns) following the Wentworth grain size classification scheme. Surface area calculations (described in detail in the text) were made from the size frequency distributions of the experimentally measured SWL-derived particles (Fig. 7; red horizontal line) and assuming geometrically equidimensional particles. The 3–4 mm-diameter detection limit of clinical CT screening (blue line) and example 250 nm-resolution CAF microscopy images are also shown. The 3 mm resolution of non-contrast computed tomography images exclude the high-frequency crystalline architecture and overall SWL-derived particle fracture geometries. Corresponding data found in Supplementary STable 3.

These estimates permit the size frequency distributions of the SWL-derived particles, as measured in the present study (Fig. 7), to be used to quantitatively estimate the impact of effective reactive surface area on post-SWL recurrence (Fig. 8). This simple approximation dramatically illustrates that a decrease in SWL-derived particle size is accompanied by an exponential increase in the total surface area available for ensuing sequential events of crystal growth and/or dissolution (*diagenetic phase transitions*)^42,78,79^ (Fig. 8). Previous experimental studies of fine-grained marine carbonate skeletal components (coral, echinoid, and algal skeletons), suggest that textural microstructure (referred to as *crystalline architecture* in the present study^38,42^), surface roughness, and grain size combine to influence total reactive surface area^45,81^. However, the dense relatively non-porous interior crystalline architecture of the CaOx kidney stone grains observed in the present study (Figs. 2, 3, 4, 5, 6) suggest that the total exterior surface area is a reasonable first-order approximation of reactive surface area in both PCNL-derived fragments and SWL-derived particles (Fig. 8).

These evaluations permit the effect of SWL-derived particle size and associated surface area to be evaluated in the context of surface- and transport-control chemical reactivity regimes^43,44^. As a result, SWL-derived particles that are below the size limit of detection by clinical non-contrast computed tomography scanners (Fig. 8) could still have an extremely high potential to serve as a nidus for crystal growth and stone recurrence^82^. In addition, these fine SWL-derived particles (Figs. 7, 8) would have a high affinity for adherence to extracellular mucus linings on renal tissues such as glycosaminoglycan hyaluronans^83^. This would serve to reduce the effectiveness of post-SWL irrigation^18^, as well decrease spontaneous passage under variable infundibulopelvic angles^16^. This also further supports the suggestion by Brain et al. that smaller SWL-derived particle size fractions should not be discounted when evaluating a patient’s risk for stone recurrence^24^. Therefore, as noted earlier, terms such as “clinically insignificant fragments” should be avoided moving forward.

The results of the present study have important implications for all applications of shock wave lithotripsy, which has been in clinical use since the 1980s and has spurred extensive controlled experimentation to better understand mechanisms of fragmentation, improve efficiency of the treatment, and reduce post-SWL stone recurrence^84^. The majority of this work has been completed on artificial composites (*begostones* or *phantom stones*) suspended in water^63,85–92^. Furthermore, other studies have shown that COM is more resistant to SWL-generated fragmentation than is COD^50^. However, this previous work used “physical properties” and did not consider the influence of original CaOx kidney stone crystalline architecture, size frequency distributions (as opposed to acoustic comminution that is based on grain averages), fracture geometries, and the influence of reactive surface area on recurrence.

Regarding fracture patterns, the original CAF- and SRAF-defined crystalline architecture of each of the PCNL-derived fragments that were subjected to ex vivo SWL treatment in the present study, creates a geometric framework within which SWL-induced fracture propagation patterns can be identified. These crystalline fabrics range from large hundreds of μm-diameter single crystals to tens of nm-diameter nanocrystals that form concentric nanolayers^93^. Further, these SWL-derived particles exhibit rectangular, pointed, concentrically spalled, and irregular particle geometries. Similar to arrow heads produced from chert (*flint*)^94^, these irregular fragments have razor sharp edges that can pierce, land on to, cut into, and damage sensitive tissues throughout the kidney, bladder, and urethra, which could further contribute to ensuing stone recurrence^28,42^.

In conclusion, the influence of SWL-derived particle size frequency distributions, fracture patterns, and reactive surface area now opens the way for many new directions of experimental research into the development of therapies meant to reduce post-SWL kidney stone recurrence. For example, factors that influence diagenetic phase transitions and reactive surface area for each Wentworth size class of SWL-derived particles can now be tested using controlled experimentation of urine-stone-microbe-renal tissue interactions within microfluidic testbeds such as the *GeoBioCell*^38–42,79^. Of specific utility is the small size of SWL-derived particles, which fit easily into GeoBioCell microfluidic flow chambers and channels, and therefore permit real-time quantitative tracking of diagenetic phase transitions controlled by a wide variety of interacting physical, chemical, and biological processes^41,93,95^. Potential examples include^39,42^: (1) real-time systematic analysis of the effect of microbiome- and host human-derived protein catalysis by promotional and inhibitory macromolecules (i.e., anionic proteins and glycosaminoglycans) on crystal aggregation and cell attachment^10,11,31,42,51,96^; (2) controls on crystal growth morphology, mineralogy, chemistry, aggregation, layering, dissolution, and recrystallization; and (3) the influence of organic acids such as citric, oxalic and formic acids excreted by specific bacteria and fungi entombed in CaOx kidney stones on stone dissolution. Furthermore, new GeoBioCell microfluidic testbeds themselves could be designed and constructed with 3D chamber printing, silica etching, and renal cell 3D printing of microfluidic flow channels in multiple configurations to simulate the actual renal hydrology, anatomy, and physiology^41,42^. In addition, effects of specific plant extracts and anti-oxidants such as hydroxy citrate can now be systematically tested with respect to post-SWL diagenetic phase transitions, cellular control, urine supersaturation, and flow rate, and their effect on CaOx kidney stone recurrence^82,97–101^.

## Materials and methods

All clinical protocols were approved by the Mayo Clinic Institutional Review Board. The experimental design and methodology applied in this study are summarized here and described in detail in the Supplementary Materials. This includes a flow chart of the systematic analyses completed in this study (Fig. 1). Kidney stone fragments were collected from one patient using standard PCNL and SPL. Medical history, standard serum labs, medication intake (e.g. citrate, thiazides, allopurinol), and comorbid conditions (e.g. diabetes mellitus, obesity, gout, hypertension, distal rental tubular acidosis, malabsorption-related conditions and diseases) were assessed from the medical record of Patient 106. Preoperative data included patient age, sex, BMI, prior surgical history, prior metabolic stone therapies, stone location based on CT scans, and stone density measurement. Metabolic panels, which included 24-h urine collection and EQUIL2-calculated supersaturation, were completed one month before and after PCNL-interventions, as well as one two years after (STab 4–5). Six ~ 4–12 mm-diameter PCNL-derived fragments (labelled 106F1-6) were collected and washed in deionized water, air dried, and imaged (SFig. 1A). Stone mineralogy was determined to be primarily calcium oxalate monohydrate (COM) using FTIR spectroscopy and CT at the Mayo Clinic Metals Laboratories (SFig. 1B). This was confirmed with high- and super-resolution microscopy and confocal Raman spectroscopy analyses at the University of Illinois Urbana-Champaign (Illinois), additionally finding trace amounts of calcium oxalate dihydrate (COD). These PCNL-derived fragments were immediately placed in a − 80 °C dry shipper dewar and transported to the Carl R. Woese Institute for Genomic Biology (IGB) at Illinois. At the time of analysis, samples were thawed for 24 h at room temperature.

Six PCNL-derived stone fragments were analyzed from a single patient, four of which were selected for this study (Fig. 1). Three-dimensional (3D) reflected-light imaging on the Zeiss AxioZoom.V16 and CT scans at 3 µm resolution on a North Star Imaging X5000 were completed on stone fragments 106F1-4 in the IGB at Illinois. The CT data was subsequently used to select the line of section and strategically orient, cut, and produce a doubly polished thin section from PCNL-derived fragment 106F3 exhibiting a complete cross-section of earliest-to-latest crystalline growth. PCNL-derived fragment 106F3 was three-dimensionally oriented, impregnated with epoxy, and made into ~ 60 µm-thick, doubly polished, uncovered thin section (106F3-1) by L. Todorov at Buehler (Chicago, IL). The remaining epoxy-embedded sample that was cut from the thin section (106F3-2), imaged on the Zeiss Axio Zoom.V16, and sent to Wagner Petrographic to be prepared into a standard-sized (24 mm × 46 mm), uncovered (no cover slip), doubly polished thin section (~ 25 µm). After receiving both 106F3-1 and 106F3-2 thin sections from Wagner Petrographic, microscopy analyses on 106F3-1 were carried out in the Microscopy and Imaging Core Facility in the Carl R. Woese Institute for Genomic Biology on a Zeiss Axio Zoom.V16, AxioScan.Z1, Axio Observer, and LSM 880 confocal system.

PCNL-derived fragment 106F1 was sacrificed for experimental standardization and PCNL-derived fragments 106F2 and 106F4 were used for the SWL studiesusing Dornier Delta^®^ III lithotripter. In preparation for SWL, two PCNL-derived fragments (106F2, 106F4) were saturated with 72-h degassed 18.2 MilliQ H_2_O for 24 h within a vacuum chamber^61^. The lithotripter was set to a rate of 90 shocks/min in increments of 100 shocks per treatment, with a coupling pressure of 4, and a power level of 3. Samples were placed in a calibration chamber attached to the SWL instrument. This configuration mimics penetration of the shock waves through human tissue and focused shockwaves at the center of a 2 mm-mesh net containing the sample. After each 100-shock treatment, SWL-derived particles sieved within the 2 mm-mesh net and SWL-derived particles that fell through the 2 mm-mesh net were separately collected and photographed. The water bath containing the fallen SWL-derived particles was extracted and filtered using a 0.47 μm mixed cellulose ester membrane filter paper within a vacuum system. The SWL-derived particles within the 2 mm-mesh net were removed, weighed, then returned for subsequent treatments. The procedure was repeated until all fragments in the 2 mm-mesh net were completely fragmented. Weight loss during SWL studies was determined ([PCNL-derived fragment weight] − [total SWL-derived particle weight]) and size frequency distributions were measured from reflected light images of loose SWL-derived particles.

SWL-derived particles from 106F2 and 106F4 were collected from the calibration container water bath after each 100-shock treatment, placed into a weigh boat, and imaged on a Zeiss Axio Zoom.V16 microscope. Image analysis was completed using Adobe Photoshop and RGB false colored (red = 210, green = 0, blue = 255) to ensure accurate grain size quantification (Fig. 4). Each image was then processed using the Zeiss AxioVision program, with the total number of fragments and measured diameters converted into XML and XLSX files. Data was evaluated on Microsoft Excel and binned according to the Wentworth grain size scale. Size frequency distributions could only be determined from loose SWL-derived particles because thin sections made from epoxied SWL-derived particles can significantly under sample the grain sizes due to the polishing-impact on the elevation of the plane of section. SWL-derived particles from 106F2 and 106F4 collected during the first SWL 100-shock treatment were embedded in epoxy blocks, polished and imaged on Zeiss AxioZoom.V16, Axio Observer, and LSM 880 Confocal microscopes. Impregnated blocks were then shipped to Wagner Petrographic Ltd. (Linden, Utah) for preparation as ~ 25 μm-thick, uncovered, doubly polished thin sections. Received thin sections of 106F2-S1 and 106F4-S1 were then imaged on the Zeiss AxioScan.Z1, LSM 880 confocal microscope with Airyscan Superresolution module and LSM 980 microscope with Airyscan Superresolution II microscopes for further analysis.

Both epoxy-embedded plugs and thin sections were imaged on a wide variety of optical modalities. A Zeiss Axio Zoom.V16 system (Carl Zeiss, Oberkochen, Germany) with a Plan-NeoFluar Z 1.0× objective and Axiocam 512 imaging device for reflective and transmitted light microscopy, including brightfield (BF) and polarization (POL) images. A Zeiss AxioScan.Z1 system with a Plan-Apochromat (10×/0.45 NA) objective and Hitachi HV-F202SCL color camera was used to capture BF, POL, and ring aperture contrast (RAC). A Zeiss Axio Observer system with a Zeiss Axiocam 506 color camera was used to capture BF, POL, and phase contrast (PC) images across a broad range of magnifications. The objectives used were Plan-Aprochromat (10×/0.45NA) Ph1 M27 DICII, Plan-Aprochromat (20×/0.80 NA) Ph2 DICII, and Plan-Aprochromat (63×/1.40 NA) Oil Ph3. For POL images, Analyzer DIC Transmitted light polarizer was utilized with the angle set to 0°. The confocal auto-fluorescence (CAF) and Airyscan super-resolution autofluorescence (SRAF) nanolayers observed in the samples were investigated and quantified using both the Zeiss LSM 880 and Zeiss LSM 980 Laser Scanning microscopes with Airyscan Super-Resolution. Tiled CAF images of merged pseudo-colored RGB channels exhibits a complete history of earliest-to-latest stone growth crystallization. All images were processed using the Zeiss Zen Blue and/or Black software to display either minimum and maximum or best-fit properties unless otherwise stated in the figure legends. In addition, red–green–blue (RGB) curves were adjusted individually or together to highlight all the crystal intensities in individual frames across the whole specimen. Where required, a non-linear gamma correction of 0.45 or 0.70 was applied to enhance faint AF crystal intensities in the same Zen program under the spline display mode property and all other corrections are presented in the corresponding figure legends. Final images were cropped, resized, and assembled using Adobe Photoshop (Adobe Systems Inc., San Jose, CA) to fit the required format.

### Disclosures

The authors have nothing to disclose except Dr. Amy E. Krambeck is a consultant for Boston Scientific.

### Ethics approval and consent to participate

This basic medical research study was reviewed and approved by the Institutional Review Board (IRB 09002083) at the Mayo Clinic. Written informed consent was obtained from all patient participants and are on file with the Mayo Clinic in Rochester, Minnesota.

## Supplementary Information

Supplementary Information.

## Data Availability

The raw microscope images and processed images are available for download from the following link: https://uofi.box.com/s/4fb9xfo41xt3r5ucgq96ryol5dtesh2f.
