# Supplementary material for: GeoBioMed perspectives on kidney stone recurrence from the reactive surface area of SWL-derived particles
Source: Sci Rep. 2022 Nov 1;12:18371. doi: 10.1038/s41598-022-23331-5 (PMC9626463; doi:10.1038/s41598-022-23331-5)
Supplement: Supplementary file 1 — Supplementary Information. [file 41598_2022_23331_MOESM1_ESM.docx]

**GeoBioMed Perspectives on Kidney Stone Recurrence from the Reactive Surface Area of SWL-Derived Particles**

Lauren G. Todorov^1,2†‡^, Mayandi Sivaguru^3†‡^, Amy E. Krambeck^4,5^, Matthew S. Lee^5^, John C. Lieske^6,7^, and Bruce W. Fouke^1,2,8,9,10†^

^1^Department of Geology, University of Illinois at Urbana-Champaign, Urbana, IL, USA.

^2^Carl R. Woese Institute for Genomic Biology, University of Illinois at Urbana-Champaign, Urbana, IL, USA.

^3^Cytometry and Microscopy to Omics Facility, Roy J. Carver Biotechnology Center, University of Illinois at Urbana-Champaign, Urbana, IL, USA.

^4^Department of Urology, Mayo Clinic, Rochester, Minnesota, USA

^5^Department of Urology, Northwestern University Feinberg School of Medicine, Chicago, Illinois, USA.

^6^Division of Nephrology and Hypertension, Mayo Clinic, Rochester, MN, USA.

^7^Department of Laboratory Medicine and Pathology, Mayo Clinic, Rochester, MN, USA.

^8^Department of Biomedical and Translational Sciences, Carle Illinois College of Medicine, University of Illinois at Urbana-Champaign, Urbana, IL, USA.

^9^Roy J. Carver Biotechnology Center, University of Illinois at Urbana-Champaign, Urbana, IL, USA.

^10^Department of Evolution, Ecology and Behavior, University of Illinois at Urbana-Champaign, Urbana, IL, USA.

†Authors contributed equally.

‡Joint first authors

Correspondence and requests for materials should be addressed to B.W.F. (email: [fouke@illinois.edu](mailto:fouke@illinois.edu)).

**Supplementary Materials**

1. **Supplementary Methods (p. 2-8)**
2. **Supplementary References (p. 9)**
3. **Supplementary Figures (p. 10-19)**
4. **Supplementary Tables (p. 20-21)**
5. **Supplementary Methods**

**Permits, IRB Approval, and Patient Consent.** This basic medical research study was reviewed and approved by the Institutional Review Board (IRB 09-002083) at the Mayo Clinic, and the outcomes of this study will positively affect the future management of kidney stone treatment. The patient provided written informed consent. Preoperative data was collected consisting of the patient age, sex, BMI, prior surgical history, prior metabolic stone therapies, stone location based on CT scan, and stone density measurement (hounsfield units). Approximately, 3-6 months after stone removal, the patient completed a metabolic evaluation for stone disease, which included 24-hour urine collection for supersaturation profile (EQUIL2). Medical history, standard serum labs, medication intake (e.g. citrate, thiazides, allopurinol), and comorbid conditions (e.g. diabetes mellitus, obesity, gout, hypertension, distal rental tubular acidosis, malabsorption-related conditions and diseases) were assessed from the medical record.

**Patient Metadata Collection.** One patient was selected for this study who formed a kidney stone primarily composed of calcium oxalate monohydrate (COM). Patient 106 was a 60-year-old female diagnosed with an episode of gross hematuria and urinary tract infection in late 2011. In 2005, the patient was status post Roux-en-Y gastric bypass for hypertension and obesity. Previous medical and surgical history of the patient includes hysterectomy for fibroids and endometriosis, degenerative joint disease, hypothyroidism, posttraumatic stress disorder, multiple personality disorder, depression, post-cholecystectomy syndrome, bladder repair for incontinence, C-section during childbirth, type 2 diabetes mellitus, abnormal adenosine sestamibi (February 2020) with a small reversible apical defect, and a laparoscopic gastric bypass (November 2005). After the gastric bypass procedure, they lost ~120lbs, although they gained 70Lbs in the following years. The patient indicated symptoms of loose stools post-surgery, especially when eating foods with higher fat content. When the patient followed a proper diet, their daily bowl movements were more consistent. The patient had one stone (1.8cm x 1.1cm) found in the left renal pelvis and one stone (8mm) with likely more smaller stones found in the left lower pole. The patient underwent percutaneous nephrolithotomy (PCNL) on February 28, 2012, and March 1, 2012, followed by the removal of some stone fragments on March 11, 2012. The left ureteral stent was removed on March 12, 2012. Follow-up CT results indicated no residual stones. The patient has been asymptomatic since the procedure with no further kidney stone issues.

**Urine Collection, PCNL Procedure, and Kidney Stone Retrieval.** One month prior to the PCNL intervention, the patient was required to submit a 24-hour urine collection sample on January 16, 2012. Stones were removed during surgery using standard PCNL procedures[^41^](#_ENREF_41)^,^[^102^](#_ENREF_102) conditions by A. E. Krambeck (author) within a sterile operating room at the Mayo Clinic in Rochester, Minnesota. The procedure began by an anesthesiologist administering pre-incision antibiotics and inducing anesthesia to the patient on a stretcher. Subsequently, a 100mL of catheterized urine sample was sterilely obtained and placed into DNA/RNAse free specimen cup by a member of the urology research team. The urine was filtered through a 0.22μm filter into three 15mL DNA/RNAse free Falcon tubes and 50mL DNA/RNAse Falcon tubes. These samples were placed in a -80°C storage freezer within 1 hour. Subsequently, Patient 106 was properly positioned and draped on the operating room (OR) table for the surgical procedure under sterile operating room conditions. The urologist obtained percutaneous access into the kidney using fluoroscopic guidance, while the urology research team obtained fluoroscopic images for 3D contextual information of the stone location prior to shock pulse lithotripsy (SPL). The kidney stones were dislodged and fragmented by the SPL probe, collected, and sent for culturing. The PCNL-derived fragments were immediately placed in a DNA/RNAse free sample cup to be stored at -80°C storage freezer within 1 hour. The patient was subsequently required to submit 24-hour urine collections one month (April 11, 2012), one year (June 24, 2013), and two years (June 30, 2014) post-PCNL intervention. The frozen urine and stone fragments were transported to the University of Illinois at Urbana-Champaign (Illinois) at -80°C Taylor-Wharton CX Series dry shipper dewar (Borehamwood, UK), and shipped to the BSL2-certified geobiology laboratory in the Carl R. Woese Institute for Genomic Biology (IGB) at Illinois. The Mayo Clinic also provided the Fouke laboratory with detailed chemical composition of the four 24-hour urine samples.

**PCNL-Derived Kidney Stone Fragment Initial Imaging.** PCNL-derived kidney stone fragments 106F1-4 and urine samples from Patient 106 were thawed for 24 hours and dried for an additional 24 hours at room temperature. All four kidney stone fragments (106F1-4) were imaged using reflected light microscopy on the Zeiss Axio Zoom.V16 microscope with a 1.0x Plan-NeoFluar Z (0.25 NA) objective, Axiocam 512 color camera, and a 1x Camera Adapter at a 7-8.2x total magnification (Carl Zeiss, Oberkochen, Germany; Figs. 3A, 4). The objective, camera, and camera adapter were consistently used for subsequent Zeiss Axio Zoom.V16 imaging in this study, unless otherwise noted.

X-ray images at 3µm-resolution were also collected on all samples using the microcomputed tomography (micro-CT) on the North Star Imaging X3000 Industrial 3D X-Ray Inspection system. PCNL-derived fragment was scanned with the following parameters: voltage of 90 kV, current of 40 µA, focal spot size 3.6µm, focal spot mode microfocus, 10 frames per second, detector pixel pitch of 0.0635 x 0.0635mm, horizontal flip, 180° rotation, tube to object distance of 12.801mm, tube to detection distance of 220.001mm, zoom factor of 16.43x, effective pixel pitch 0.00739mm, 2520 projections, voxel size of 3.9µm, and geometric unsharpness of 15.4x. PCNL-derived fragment 106F2 was scanned with the following parameters: voltage of 90 kV, current of 40 µA, focal spot size 3.6µm, focal spot mode microfocus, 10 frames per second, detector pixel pitch of 0.0635 x 0.0635mm, horizontal flip, 180° rotation, tube to object distance of 12.799 mm, tube to detection distance of 220.001 mm, zoom factor of 16.42x, effective pixel pitch 0.007388 mm, 60 projections, voxel size of 3.9µm, and geometric unsharpness of 15.4x. PCNL-derived fragments 106F3 and 106F4 were scanned with the following parameters: voltage of 90 kV, current of 40 µA, focal spot size 3.6µm, focal spot mode microfocus, 10 frames per second, detector pixel pitch: 0.0635 x 0.0635mm, horizontal flip, 180° rotation, tube to object distance of 17.729 mm, tube to detection distance of 220.76 mm, zoom factor of 12.02x, effective pixel pitch 0.01023 mm, 2520 projections, voxel size of 5.3µm and geometric unsharpness of 11x. This permitted the generation of several thousand images per core. Raw data images were converted to Tiff files at both 8-bit and 16-bit scales for visualization. The images were rendered in 3D and virtual sections were made in the native system software and resized using NIH Image J Software (https://imagej.nih.gov/ij/).

**PCNL-Derived Stone Fragment Experimental Preparation.** PCNL-derived fragment 106F1 was sacrificed for experiment standardization. PCNL-derived fragments 106F2 and 106F4 were initially weighed, then saturated for 72 hours with 24-hour degassed 18.2 MilliQ H2O within a vacuum chamber[^61^](#_ENREF_61)^,^[^103^](#_ENREF_103). The samples were then rinsed for 10 minutes in a fresh beaker of 24+ hour degassed 18.2 MilliQ water using forceps to remove any dissociated particles from the stones. The stones were then gently tapped onto a Kim wipe and transferred to a weigh boat to record the final weight prior to the SWL experiment. The 72-hour-H_2_O saturated sample was then used for the SWL fragmentation experiment within the hour.

**Dornier ESWL Instrument Preparation.** The Dornier Delta® III urological workstation instrument (https://www.dornier.com/products/) containing the ESWL was donated by Dornier MedTech to the Fouke Lab at the UIUC and was used for this study. Prior to initiating the experiment, the instrument was warmed up and LithoClear Lithotripsy gel was strategically (<https://www.coneinstruments.com/lubrication/p/LithoClear-Lithotripsy-Gel/>) mounted on the bellow with limited air bubbles. This was done by holding the gel container at the center of the bellow with little movement to create a large mound, then using a spatula to remove air bubbles and evenly distribute the gel across the top of the bellow.

This study used the provided calibration container for the SWL fragmentation experiment since it was designed to mimic the tissue-kidney structure with a direct shockwave focal point at the center, 2mm-mesh net containing the sample[^88^](#_ENREF_88). The container was attached to the SWL, and the bellow coupling pressure was shifted from 5 to 4 to properly adhere the gel onto both the bellow and test container surface. The presence of bubbles within the gel can interfere with the shock penetration. If bubbles were present, the edge of the bellow was pressed downward to move the trapped air out from the gel, or the instrument was left for running to allow the radiating heat to remove any remaining bubbles. Subsequently, 1000mL of 18.2 MilliQ water was poured into the test container water bath, completely submerging the protruding 2mm-mesh net. The 72-hour-H2O saturated PCNL-derived fragment was then placed directly within the center of the 2mm-mesh net using forceps and images by phone were immediately obtained of T-0 (Fig. 4A, D; SFig. 4).

**SWL-Derived Particle Experimental Design.**  In this experiment, the SWL instrument was set to a shock rate of 90 shocks/min, coupling pressure of 4, power level of 3, with increments of 100 shocks per treatment over a duration of less than 1 hour. The dissolved oxygen concentration of the water in the chamber was not measured[^103^](#_ENREF_103). After each 100-shock SWL treatment, the SWL-derived particles sieved within the 2mm-mesh net and the SWL-derived particles small enough to pass through the 2mm-mesh net and into the water bath were imaged by phone, then separately collected and processed.

*Stone Fragments Collected from the 2-mm Nylon Mesh Basket.* After each 100-shock SWL treatment, the SWL-derived particles sieved within the 2mm-mesh net were collected using forceps, tapped onto a Kim wipe, placed into a weigh boat, imaged by phone, and weighed. SWL-derived particles that fell through during this collection were obtained at the bottom of the calibration container with forceps or a pipette. Subsequently, an additional 1000mL of 18.2 MilliQ water was poured into the calibration container water bath and the SWL-derived particles were carefully placed back into the 2mm-mesh net for another treatment cycle. At this point, if any small SWL-derived particles fell through, they may have been left at the bottom of the container. Although, an electric pipette and forceps were utilized to capture larger SWL-derived particles and transfer them back into the center 2mm-mesh net prior to initiating the next 100-shock treatment. A final representative image of the remaining SWL-derived particles within the 2mm-mesh net was obtained by personal cell phone. The process was repeated until no SWL-derived particles were detected in the center 2mm-mesh net. For analysis, the weights obtained after each 100-shock SWL treatment were processed to generate representative graphical outputs using Microsoft Excel (Fig. 7A; STab. 1).

*Stone Fragments Collected from the Water Bath*. After each 100-shock SWL treatment, the calibration container water bath was drained in 120mL increments using the Med Lab Supply hand vacuum pump with pressure gauge ([https://www.medical-and-lab-supplies.com/hand-vacuum-pump-with-pressure-gauge.html](https://www.medical-and-lab-supplies.com/hand-vacuum-pump-with-pressure-gauge.html?utm_campaign=Smart+Shopping+Test&utm_source=adwords&utm_term=&utm_medium=ppc&hsa_acc=2462377349&hsa_mt=&hsa_ad=346646813178&hsa_kw=&hsa_ver=3&hsa_src=u&hsa_grp=69333253869&hsa_tgt=pla-370435928838&hsa_cam=1854631343&hsa_net=adwords&gclid=Cj0KCQjw8fr7BRDSARIsAK0Qqr5rcJsnQEdf6NTsA6hA1mG_mP0Psai1_W4x5rMx7dDrCm_Ofsj7JggaAp9QEALw_wcB)) into a 1000mL beaker. An Accu-Jet Pro electronic pipette controller (<https://www.brandtech.com/product/accu-jet-pro/>) attached to a 10mL tip, with 1cm of the tip cutoff, was used to collect visible, broken fragments within the test container. The beaker with the water bath containing the SWL-derived particles was left for 10 minutes at an angle, allowing for the SWL-derived particles to settle. Subsequently, the 1000mL solution was filtered through a 500mL vacuum filtration system using GE Healthcare Life Sciences Whatman 0.47μm mixed cellulose ester membrane filter paper. The filter paper was then transferred into a labeled petri dish for imaging and quantification. This procedure was repeated with each 100-shock SWL treatment.

**Reflected Light Imaging and SWL-Derived Particle Grain Size Quantification and Classification.** Loose SWL-derived particles from the water bath collected from each 100-shock SWL treatment were placed within an orange rubber ring on a weigh boat to be imaged, quantified, and characterized by grain size. Complete 4 x 4 stitched tiled images (each comprising 14443 x 9626 pixels; 63.96 mm x 42.64 mm) were obtained using reflective light microscopy on the Zeiss Axio Zoom.V16 microscope with a total magnification of 7x. Subsequently, all loose SWL-derived particles from each 100-shock SWL treatment were carefully transferred into individually labelled Eppendorf tubes for storage. The images were then uploaded into Adobe Photoshop and each loose SWL-derived particle was carefully outlined by hand and filled with one selected color (RGB of 210 for red, 0 for green, and 255 for blue) to ensure more accurate selection during grain size quantification (Fig. 4B, E). Each image had a xy resolution of 4.429μm/px. Each individual image was uploaded into the Zeiss AxioVision program to be processed using a brightness of -0.56, contrast of 1.12, gamma of 1.00, sigma of 32, shading correction brightness of -185, edge enhancement delineate threshold of 25, edge enhancement delineate size of 3, deletion of artifact minimum of 0 and maximum of 90753696 with selected fill holes, and automatic object separation mode of watershed and settings tolerance of 2. The RGB selection range for the loose SWL-derived particles from PCNL-derived fragment 106F2 were 204-223 for red, 0-104 for green, and 192-255 for blue. The RGB selection range for the loose SWL-derived particles from PCNL-derived fragment 106F4 fragments were 207-223 for red, 0-104 for green, and 216-255 for blue. Using these settings, a final output containing the total number of stones and each measured (largest) diameter was obtained and converted into an XML and XLSX file. Subsequently, this data was characterized and visually graphed using Microsoft Excel according to the Wentworth grain size scale[^48^](#_ENREF_48). Size frequency distributions could only be determined from loose SWL-derived particles because thin sections made from epoxied SWL-derived particles can significantly under sample the grain sizes due to the polishing-impact on the elevation of the plane of section.

**Epoxy Plug Embedding of Loose SWL-Derived Particle Subset.** Loose SWL-derived particles collected from the first 100-shock SWL treatment for both PCNL-derived fragments 106F2 (106F2-S1) and 106F4 (106F4-S1) contained the largest number of particles, thus, were selected for epoxy embedding. The loose SWL-derived particle groups, 106F2-S1 and 106F4-S1, were carefully placed onto a Buehler MET Grip Liner Adhesive within a 1in x 1in Buehler SamplKup. Properly mixed Buehler EpoThin 2 media (22.6g hardener and 50.0g resin) without air bubbles was poured onto the adhesive liner within the SamplKup, then placed into the Buehler SimpliVac machine for infiltration at 1 cycle for 30 seconds at -25.7inHg. The samples were then placed in a heater overnight to cure. The following day, the adhesive liner was slowly removed, and the hardened epoxy-embedded plugs were dislodged from the SamplKup. The epoxy-embedded SWL-derived particle samples of 106F2-S1 and 106F4-S1 were polished on the fragment-exposed surface using the Buehler AutoMet 250 Polisher in the following sequence: (1) Buehler 800 Grit (5μm) or 600 Grit to grind into the stone and remove epoxy; (2) Buehler 3μm diamond paste on a Buehler Trident Pad; and (3) Buehler Alumina paste on a Buehler MicroCloth. The epoxy plugs were then submerged in a beaker of ethanol and placed in the Buehler UltraMet 2003 Sonic Cleaner containing Simple Green All Purpose Cleaner diluted with water. This procedure was completed by L. Todorov (author) at Buehler in Lake Bluff, IL. The epoxy-embedded 106F2-S1 and 106F4-S1 samples were imaged on the Zeiss Axio Zoom.V16, Axio Observer.Z1, and Zeiss LSM 880 microscopes before being sent to Wagner Petrographic Ltd. in Linden, Utah to be converted into standard-sized (24mm x 46mm), uncovered (no slip), doubly polished thin sections with a thickness of ~25μm.

**Preparation of Thin Sections 106F3-1 and 106F3-2.** PCNL-derived kidney stone 106F3 was selected to be thin sectioned. A 1in x 1in Buehler SamplKup was assembled using the Buehler Release Agent and filled with properly mixed EpoThin 2 media. When the epoxy hardened, the PCNL-derived fragment 106F3 was strategically oriented with mounting clay using the micro-CT data from the North Star Imaging X3000 Industrial 3D X-Ray Inspection system onto the hardened epoxy, selecting a line of section (cut) with the optimal orientation for the visualization of the crystal growth axis within the complete thin section. Additional epoxy was carefully added using a syringe until the selected line of section was reached and the epoxy on the partially submerged PCNL-derived fragment 106F3 was allowed to cure overnight. The following day, the sample was removed from the SamplKup and the dried-epoxy meniscus was grinded off using sandpaper. The epoxy-embedded PCNL-derived fragment 106F3 was cut at the selected orientation (epoxy surface) using the Buehler IsoMet Low Speed Precision Cutter with the Buehler IsoMet 10LC Precision Blade and 1.75in aluminum flange set. The non-epoxy embedded PCNL-derived fragment 106F3 portion that detached from the embedded plug was collected and stored for further decalcification experiments. The epoxy-embedded plug was then returned to the SamplKup, placed in the SimpliVac system with loaded EpoThin 2 epoxy, completely submerged with epoxy while the system was run for 10 cycles at 1 minute with -25.4inHg, and left to cure overnight to allow epoxy to infiltrate the stone through the pore space.

The following day, the epoxy-embedded plug containing the PCNL-derived fragment 106F3 was removed from the SamplKup and prepared for polishing by placing it within the Buehler central force specimen plate (5.1in and 6.1in) with two blank epoxy plugs, using the Buehler Specimen Loading Fixture. The epoxy-embedded plug containing PCNL-derived fragment 106F3 was polished on the surface closest to the stone using the Buehler AutoMet 250 Polisher in the following sequence: (1) Buehler Diamond Grinding Red (75μm) Magnetic Disk; (2) Buehler Diamond Grinding Yellow (35μm) Magnetic Disk; (3) Buehler PSA Backed MicroCut 800 [P1500] (13μm) disk; (4) Buehler MetaDi Diamond Green Paste (3μm) on a Buehler Trident Polishing Cloth with MetaDi Fluid; and (5) Buehler Alumina paste on a Buehler MicroCloth. The sample was then submerged in a beaker of ethanol and placed in the Buehler UltraMet 2003 Sonic Cleaner containing Simple Green All Purpose Cleaner diluted with water. A standard sized (24mm x 46mm) microscope slide was placed on a rectangular piece of MetGrip Liner Adhesive and properly mixed EpoThin 2 media was evenly coated in the center using a mixing stick. The 106F3 epoxy-embedded plug was placed on the slide and left to cure overnight using the Buehler PetroBond on a hot plate at 35-40°C.

A thin section containing the PCNL-derived fragment 106F3 epoxy-embedded plug was cut ~500 µm from the glass slide using the Buehler IsoMet High Speed Pro using the IsoMet 15HC Blade and the 1.38in (35mm) aluminum flange set at settings 5 mm/min, cutting depth of 40mm, 4000 RPM, serial cut off and smart cut off selected, coolant set to off, blade dress set to off, and manual dress option set to off. The thin section was named 106F3-1, while the remaining epoxy-embedded sample was named 106F3-2. The 106F3-1 thin section was then placed into the Buehler Stage Micrometer using Buehler Lapping Oil and polished to ~60µm thickness using the AutoMet 250 Polisher in the following sequence: (1) Buehler Purple Diamond Grinding (55μm) Disk; (2) Buehler Terra Diamond Grinding (9μm) Disk; (3) Buehler MetaDi Diamond Green Paste (3μm) on a Buehler Trident Polishing Cloth with MetaDi Fluid; and (4) Buehler Alumina paste on a Buehler MicroCloth. Some regions of the 106F3-1 were plucked during thin section polishing and preparation. This procedure was completed by L. Todorov (author) at Buehler in Lake Bluff, IL. The 106F3-1 thin section was imaged on the Zeiss Axio Zoom.V16, AxioScan.Z1, Axio Observer.Z1, and Zeiss LSM 880 microscopes before being sent to Wagner Petrographic for final polishing to ~25μm thickness. The 106F3-2 embedded sample was imaged on the Zeiss Axio Zoom.V16 before also being sent to Wagner Petrographic to be converted into standard-sized (24mm x 46mm), uncovered (no slip), doubly polished thin sections with a thickness of ~25μm. The received thin sections were then imaged on the Zeiss AxioScan.Z1 and LSM 980 Confocal microscopes.

**Bright-Field (BF), Polarization (POL), Phase Contrast (PC), and Ring Aperture Contrast (RAC) Microscopy.** A wide variety of optical modalities were applied in the present study to image the kidney stone thin sections[^41^](#_ENREF_41)^,^[^42^](#_ENREF_42)^,^[^79^](#_ENREF_79)^,^[^104-110^](#_ENREF_104). These include BF, POL, PC, and RAC as described before[^19^](#_ENREF_19)^,^[^41^](#_ENREF_41)^,^[^78^](#_ENREF_78)^,^[^79^](#_ENREF_79)^,^[^107^](#_ENREF_107)^,^[^108^](#_ENREF_108). We have used a suite of Zeiss instruments with the appropriate optical magnifications to render an ultimate spatial resolution of 250nm. These include: (1) Zeiss Axio Zoom.V16 microscope with a DL 450 LED light source base, Axiocam 512 color camera, and a 1.0x Plan-NeoFluar Z NA 0.25 objective for both reflective and transmitted light microscopy (BF and POL); Zeiss AxioScan.Z1 whole slide scanner system with a TL LED lamp, Hitachi HV-F202SCL camera, and a Plan Aprochromat 10x/ 0.45 NA objective (for BF, POL, and RAC); and (3) Zeiss Axio Observer.Z1 microscope with TL Halogen lamp, Zeiss Axiocam 506, Analyzer DIC Transmitted Light Reflector with polarizer set to 0° (for POL), Plan Aprochromat 10x/0.45 NA Ph1 M27 objective, Plan Aprochromat 20x/0.80 NA Ph2 DICII objective, and Plan Aprochromat 63x/1.40 Oil Ph3 objective (for BF, POL, and PC). Most microscopy in this study (some performed at the Zeiss North American Head Quarters, Pleasanton, CA) was completed in the Microscopy and Imaging Core Facility of the IGB on the Illinois campus. This was the first North American laboratory selected as the Zeiss Labs@Location Partner by Carl Zeiss LLC, a recognition that facilitated the integrated assemblage of all the microscopy modalities applied in this study.

**Confocal Auto-Fluorescence (CAF) and Airyscan Super-Resolution AF (SRAF).** The fracture patterns were characterized, and specific locations were selected to be further investigated using the Zeiss LSM 880 and LSM 980 Scanning microscopes with Airyscan Super-Resolution[^110^](#_ENREF_110). These instruments have a resolution significantly greater than all other currently available confocal diffraction-limited microscopy techniques by a factor of 1.7, to provide a spatial resolution of ~140nm[^41^](#_ENREF_41)^,^[^104^](#_ENREF_104)^,^[^106^](#_ENREF_106)^,^[^109^](#_ENREF_109)^,^[^110^](#_ENREF_110). We have used these systems in both super-resolution autofluorescence (SRAF; scan zoom ≥ 2; Figs 3A,D, E, 6H; SFig. 10B) and confocal auto fluorescence (CAF; scan zoom < 2; Figs. 3B, C, F, G, H, 5, 6A-G; SFig. 10A, C) modes. Excitation and emission wavelengths that were collected on the LSM 880 include: 405nm excitation (emission collected at 450nm; blue), 488nm excitation (emission collected at 525nm; green), and 561nm excitation (emission collected at 593nm; red). Excitation and emission wavelengths that were collected on the LSM 980 include: 353nm excitation (emission collected at 465nm; blue), 492nm excitation (emission collected at 517nm; green), and 577nm excitation (emission collected at 603nm; red). On the LSM 880, an internal photo-multiplier tube detector was used to scan selected locations using a Plan-Aprochromat 63x/1.4 NA Oil immersion DIC M27 objective. On the LSM 980, the objectives used were the Plan Aprochromat 10x/0.45 NA M27 and Plan Aprochromat 20x/0.8 NA M27. Simultaneously, transmitted-light images were obtained using a 561nm laser transmission photomultiplier (T-PMT) detector on the same system (Figs. 3B, 4A, C, E, G; Sup Figs. 6, 7, and 8). The main beam splitters (MBS used in the light path of the detector were MBS 488/561 for visible and MBS 405 for invisible light paths for SRAF, CAF, and T-PMT. SRAF and CAF also the dichronic beam splitter SPS SP 615. Raw data images from 32 Airyscan detectors were processed using the Airyscan processing modality using the auto-2D to obtain a super-resolution image.

**Image Adjustments, Analysis, Preparation, and Presentation.** All images were processed using the Zeiss Zen Blue and/or Black software to display either minimum and maximum or best-fit properties[^111^](#_ENREF_111) unless otherwise stated in the figure legends. In addition, red-green-blue (RGB) curves were adjusted individually or together to highlight all the crystal intensities in individual frames across the whole specimen. Where required, a non-linear gamma correction of 0.45 or 0.70 was applied to enhance faint AF crystal intensities in the same Zen program under the spline display mode property and all other corrections are presenting in the corresponding figure legends. All raw data was stored in native CZI and then exported as TIF files with no compression after adjusting the display properties. Final images were cropped, resized, adjusted for optimal brightness and contrast, and assembled using Adobe Photoshop (Adobe Systems Inc., San Jose, CA) to fit the required format and optimal visualization.

**Raman Spectroscopy of Thin Section 106F3-1.** A WITec Alpha 300RA AFM-Raman system was used to investigate the composition of thin section 106F3-1. The UHTS 600 VIS spectrometer associated with the AFM-Raman system was used for better sensitivity and peak separation. Two large contextual areas using high magnification 20-100x objectives were scanned and imaged. The large image scans were stitched automatically using the corresponding software. To prevent autofluorescence, a 532nm laser was used with 0.1s integration time and 150-250 points per line setting. Raman images were converted to pseudo-color-coded images and overlaid correlatively on transmitted light BF images for easy comparison. Raman spectra were background subtracted prior to final analysis using the Project 5 WITec software. The acquired Raman peaks were correlated and compared with previously published Raman peaks for human kidney stones[^39^](#_ENREF_39)^,^[^42^](#_ENREF_42)^,^[^112-114^](#_ENREF_112).

1. **Supplementary References**

102 Patil, A. V. A Novel 5-Part Percutaneous Access Needle With Glidewire Technique (5-PANG) for Percutaneous Nephrolithotomy: Our Initial Experience. *Urology* **75**, 1206-1208, doi:10.1016/j.urology.2009.11.027 (2010).

103 Sankin, G. N. *et al.* Elimination of cavitation-related attenuation in shock wave lithotripsy. doi:10.1063/1.4977638 (2017).

104 Kolossov, V. L. *et al.* Airyscan super-resolution microscopy of mitochondrial morphology and dynamics in living tumor cells. *Microscopy Research and Technique* **81**, 115-128, doi:10.1002/jemt.22968 (2018).

105 Singh, R. *et al.* Real rock-microfluidic flow cell: A test bed for real-time in situ analysis of flow, transport, and reaction in a subsurface reactive transport environment. *Journal of Contaminant Hydrology* **204**, 28-39, doi:10.1016/j.jconhyd.2017.08.001 (2017).

106 Sivaguru, M., Fried, G. A., Miller, C. A. H. & Fouke, B. W. Multimodal Optical Microscopy Methods Reveal Polyp Tissue Morphology and Structure in Caribbean Reef Building Corals. *Journal of Visualized Experiments*, doi:10.3791/51824 (2014).

107 Sivaguru, M., Mander, L., Fried, G. & Punyasena, S. W. Capturing the Surface Texture and Shape of Pollen: A Comparison of Microscopy Techniques. *PLoS ONE* **7**, e39129, doi:10.1371/journal.pone.0039129 (2012).

108 Sivaguru, M. *et al.* Comparative performance of airyscan and structured illumination superresolution microscopy in the study of the surface texture and 3D shape of pollen. *Microscopy Research and Technique* **81**, 101-114, doi:10.1002/jemt.22732 (2016).

109 Urban, M. A., Barclay, R. S., Sivaguru, M. & Punyasena, S. W. Cuticle and subsurface ornamentation of intact plant leaf epidermis under confocal and superresolution microscopy. *Microscopy Research and Technique* **81**, 129-140, doi:10.1002/jemt.22667 (2016).

110 Huff, J. The Airyscan detector from ZEISS: confocal imaging with improved signal-to-noise ratio and super-resolution. *Nature Methods* **12**, i-ii, doi:10.1038/nmeth.f.388 (2015).

111 Cromey, D. W. Avoiding Twisted Pixels: Ethical Guidelines for the Appropriate Use and Manipulation of Scientific Digital Images. *Science and Engineering Ethics* **16**, 639-667, doi:10.1007/s11948-010-9201-y (2010).

112 Takasaki, E. Carbonate in Struvite Stone Detected in Raman Spectra Compared with Infrared Spectra and X-Ray Diffraction. *International Journal of Urology* **3**, 27-30, doi:10.1111/j.1442-2042.1996.tb00625.x (1996).

113 Balan, V. *et al.* Vibrational Spectroscopy Fingerprinting in Medicine: from Molecular to Clinical Practice. *Materials* **12**, 2884, doi:10.3390/ma12182884 (2019).

114 Castiglione, V. *et al.* Raman chemical imaging, a new tool in kidney stone structure analysis: Case-study and comparison to Fourier Transform Infrared spectroscopy. *PLOS ONE* **13**, e0201460, doi:10.1371/journal.pone.0201460 (2018).

1. **Supplementary Figures**

**
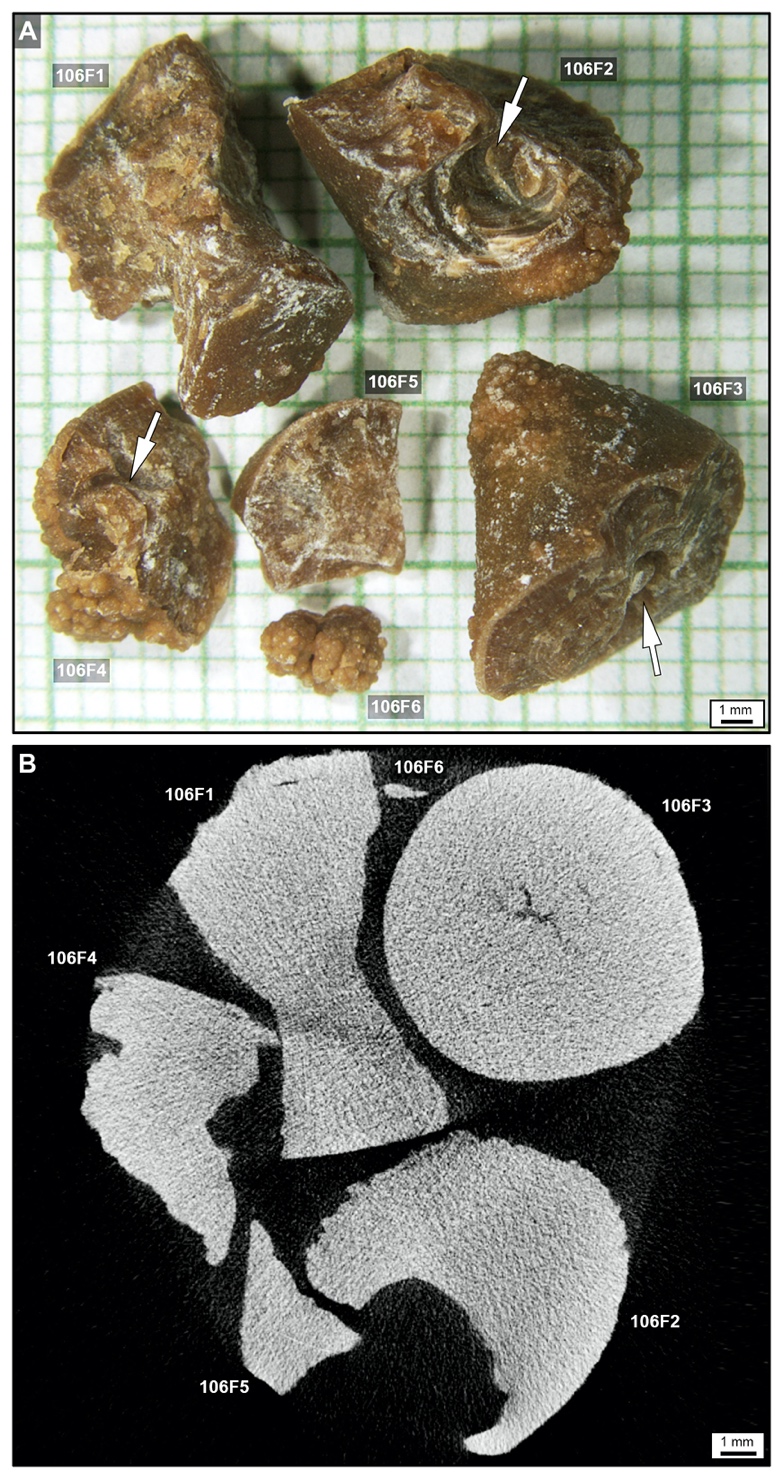
**

**Supplementary Figure 1.** Six PCNL-derived fragments from Patient 106 from the Mayo Clinic. The major axis (longest diameter) for each fragment is: 106F1, 11.47 mm; 106F2, 11.51 mm; 106F3, 11.67 mm; 106F4, 8.36 mm; 106F5, 6.31 mm; and 106F3, 4.08 mm. (A) RL image of PCNL-derived fragments 106F1-6 taken at the Mayo Clinic. (B) Micro-CT image of PCNL-derived fragments 106F1-6 taken at the Mayo Clinic. PCNL-derived fragments 106F1-4 were used in the present study. White arrows show notches resulting during PCNL SPL procedure.


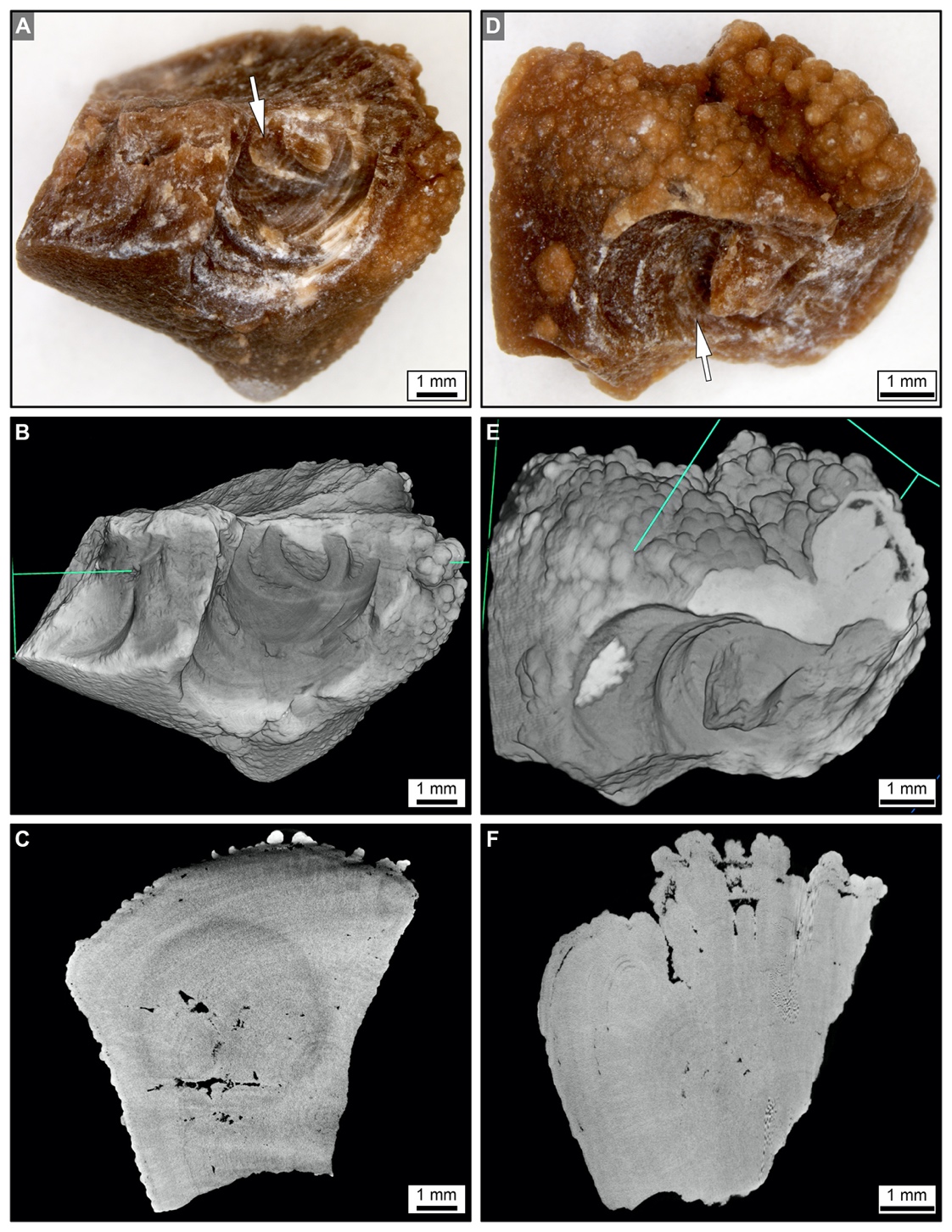


**Supplementary Figure 2.** Three-dimensional (3D) external morphology and two-dimensional (2D) internal crystalline architecture of PCNL-derived fragments 106F2 (**A**-**C**) and 106F4 (**D**-**F**) prior to experimentation. (**A, D**) 3D RL image of the entire stone showing notches (white arrows) resulting during PCNL SPL procedure. (**B**, **E**) 3D micro-CT of the external surface rendering of the entire stone. (**C**, **D**) 2D virtual micro-CT cross-section of the internal crystalline structure. Corresponding figure of stones during experimentation presented in Figure 4 and Supplementary Figure 4.


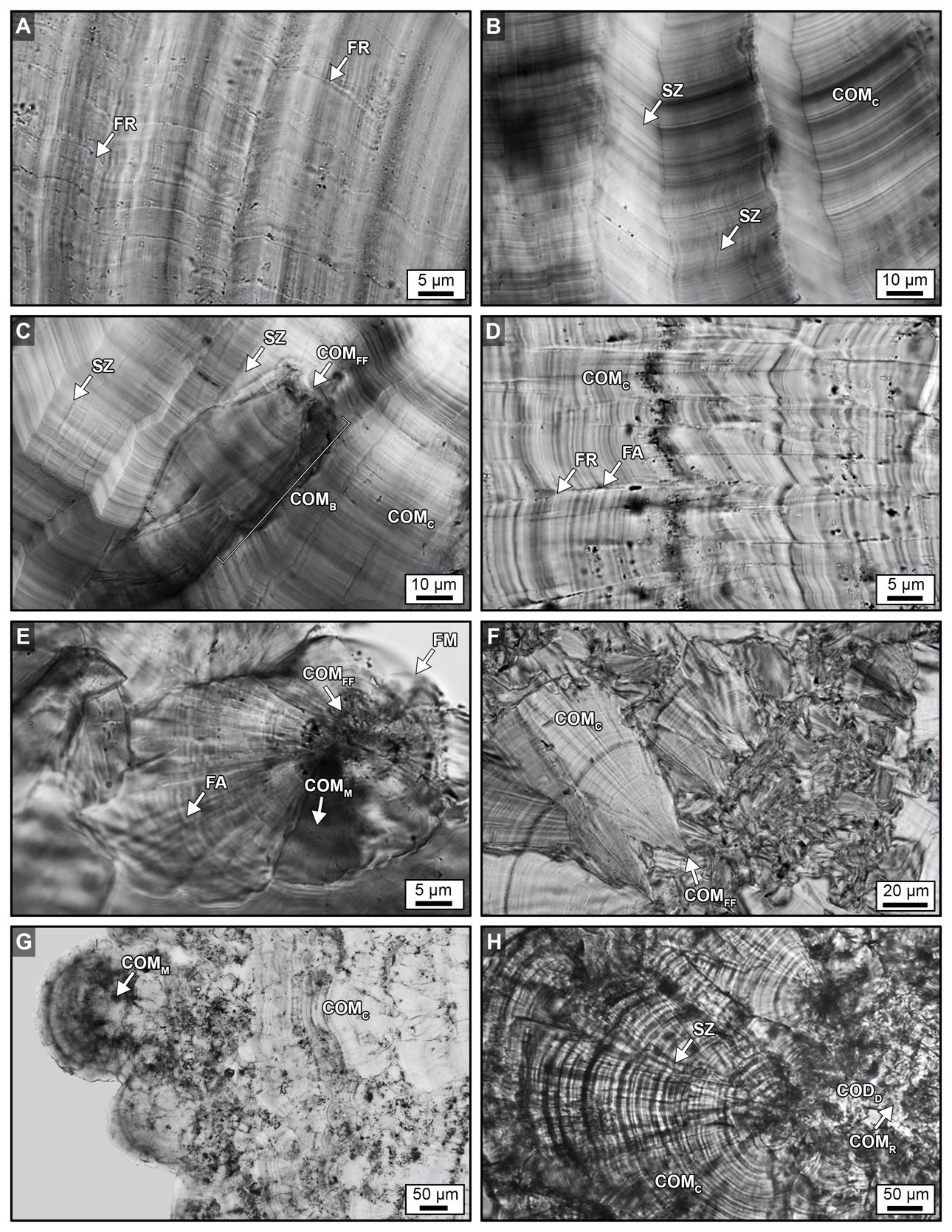


**Supplementary Figure 3.** Original crystalline architecture of CaOx PCNL-derived fragments. (**A-H**) T-PMT images. Labels indicate: FR, fracture; FA, fault; SZ, sector zone; COM_FF_, free-floating COM; COM_C_, COM cortex; COM_B_, bundles of COM radiating from a COM_FF_; COM_M_, mimetic replacement COM; COM_R_, replacement COM; and COD_D_, dissolved COD. Image locations shown in Figures 2B-D and 4C and corresponding SRAF and CAF images presented in Figure 3.


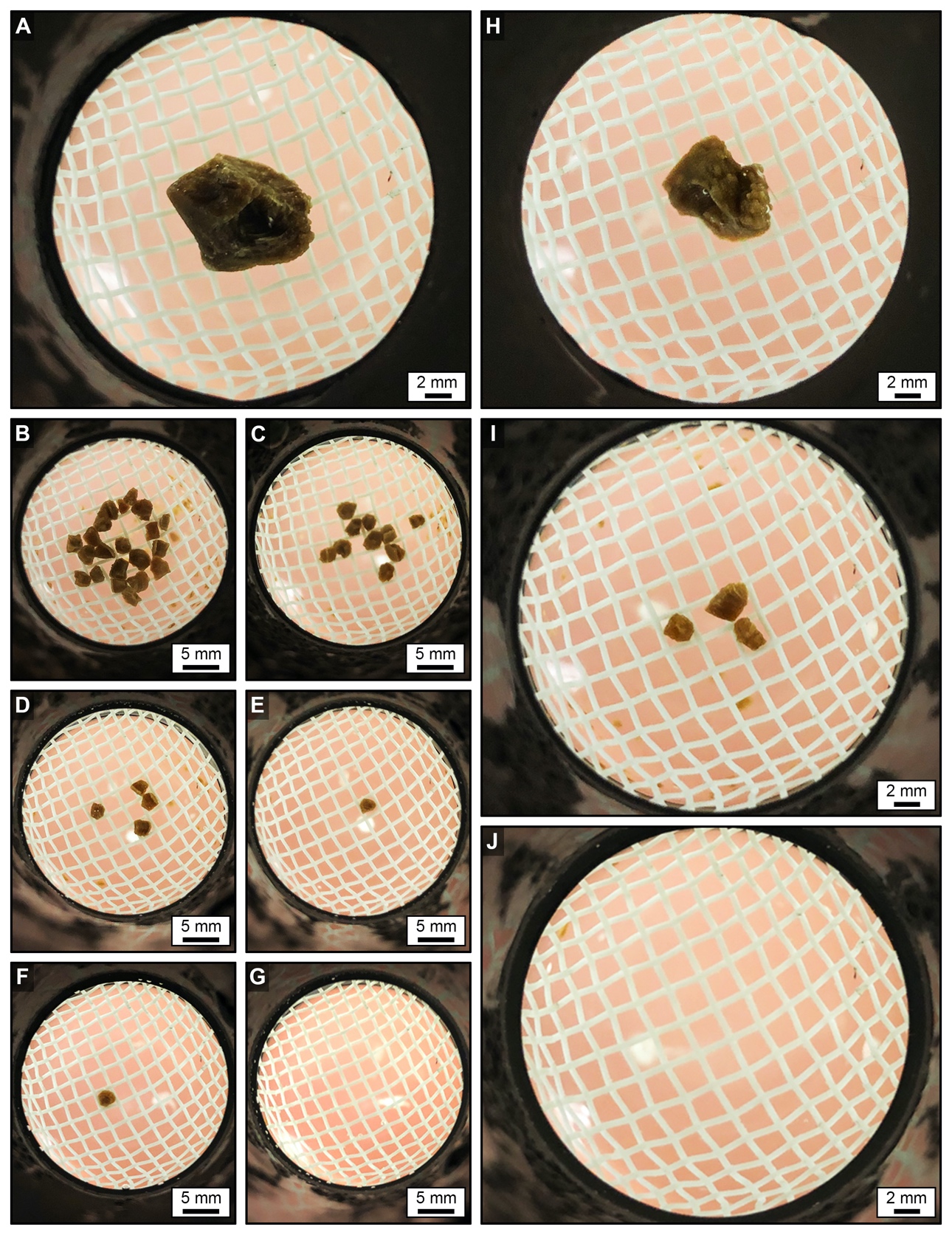


**Supplementary Figure 4.** Weight changes of SWL-derived particle groups 106F2 (**A**-**G**) and 106F4 (**H**-**J**) suspended within 2mm-mesh net basket. (**A**, **H**) RL images of PCNL-derived fragment prior to experimentation. (**B**, **C**, **D**, **E**, **F**, **G**, **I**, **J**) RL images of SWL-derived particles during experimentation. Corresponding images presented in Figure 4A, D and Supplementary Figure 2 and data is presented in Figure 7A and Supplementary Table 1.


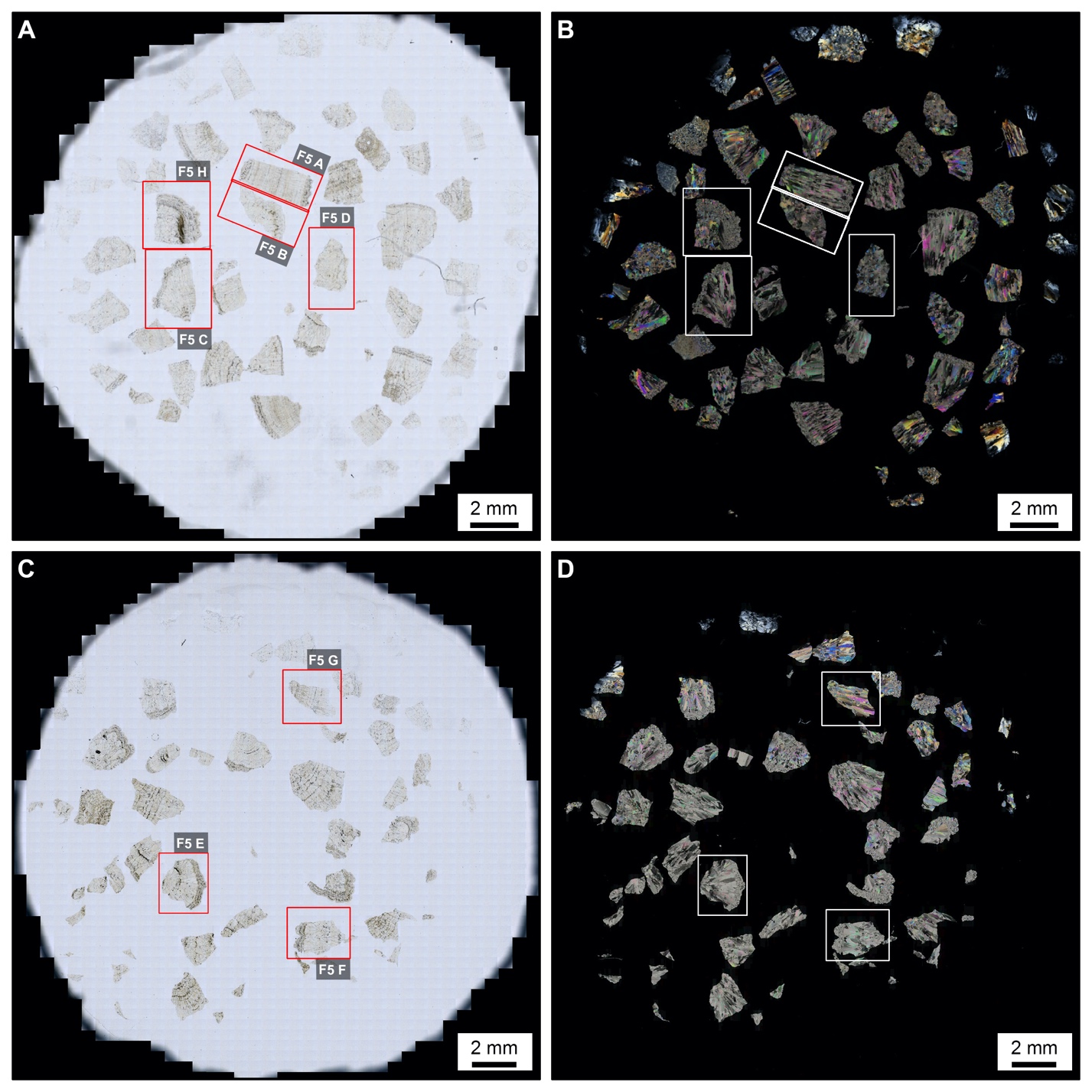


**Supplementary Figure 5.** Geometries of SWL-derived particles from groups 106F2-S1 and 106F4-S1 (shown in Fig. 5) that were thin sectioned. (**A, C**) BF images. (**B, D**) POL images. Corresponding CAF, BF, and POL images presented in Figure 5 and Supplementary Figures 6 and 7.


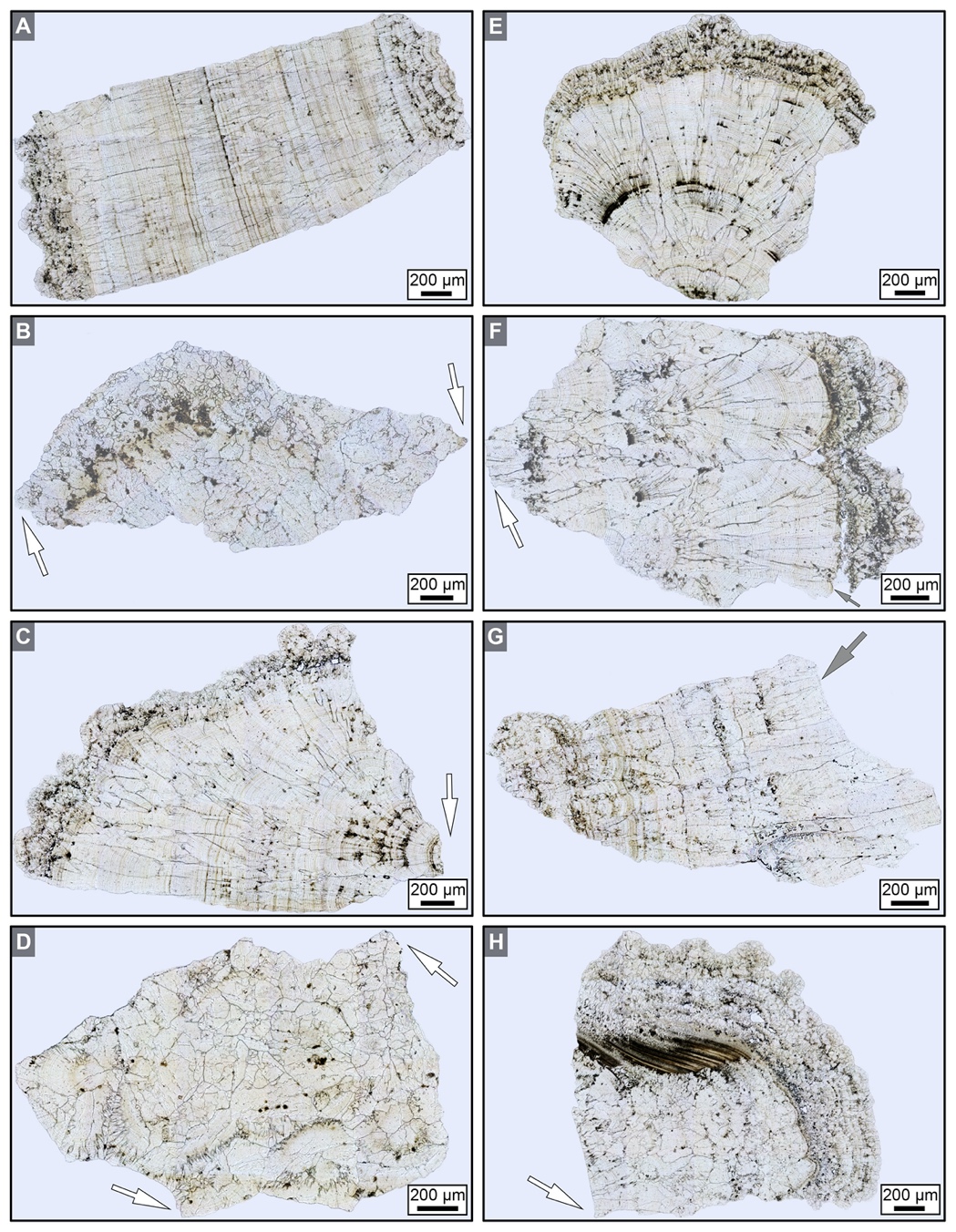


**Supplementary Figure 6.** Geometries of SWL-derived particles from groups 106F2-S1 and 106F4-S1 (Fig. 4). These detailed crystal growth structures and fracture patterns, observed in thin section, are analogous to those observed on the polished epoxy plugs (Figs. 3, 6). The background has been removed from around, but not within each fragment, and replaced with a grey background. (**A**-**H**) BF images showing SWL fracture geometries that crosscut the original CaOx crystalline architecture. SWL shock fractures propagate at perpendicular and oblique angles with respect to the original crystalline architecture, often converging to form angles of 60-120° (**B**, **C**, **D**, **F**, **H**, white arrows). Spalling along concentric crystalline layering is also observed (**F**, **G**, grey arrows). SWL-derived particle locations shown in Figure 4C, F and Supplementary Figure 5 and corresponding CAF and POL figures presented in Figure 5 and Supplementary Figures 7.


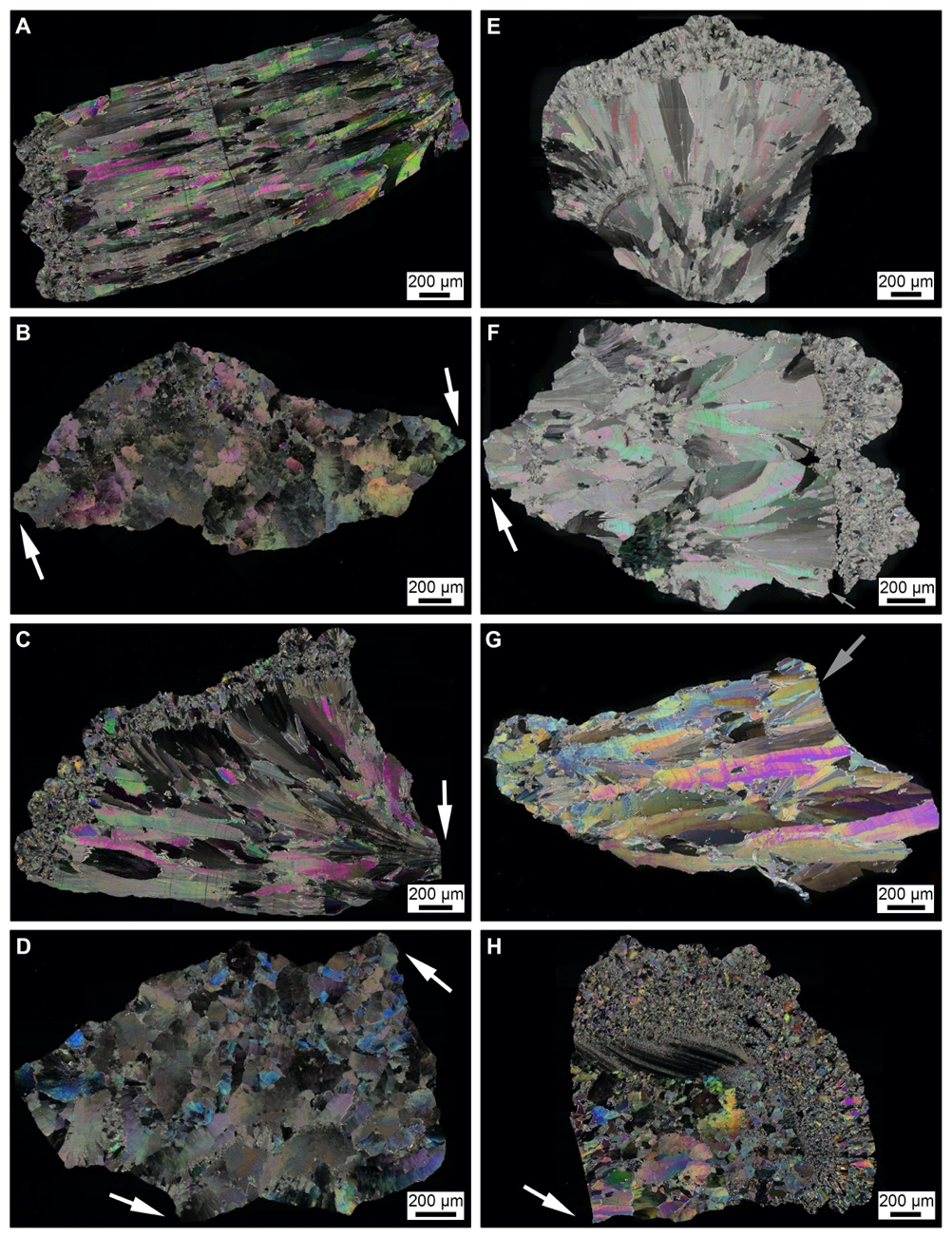


**Supplementary Figure 7.** Geometries of SWL-derived particles from groups 106F2-S1 and 106F4-S1 (Fig. 4). These detailed crystal growth structures and fracture patterns, observed in thin section, are analogous to those observed on the polished epoxy plugs (Figs. 3, 6). The background has been removed from around, but not within each fragment, and replaced with a black background. (**A**-**H**) POL images showing SWL fracture geometries that crosscut the original CaOx crystalline architecture. SWL shock fractures propagate at perpendicular and oblique angles with respect to the original crystalline architecture, often converging to form angles of 60-120^o^ (**B**, **C**, **D**, **F**, **H**, white arrows). Spalling along concentric crystalline layering is also observed (**F**, **G**, grey arrows). SWL-derived particle locations shown in Figure 4C, F and Supplementary Figure 5 and corresponding CAF and BF figures presented in Figure 5 and Supplementary Figures 6.


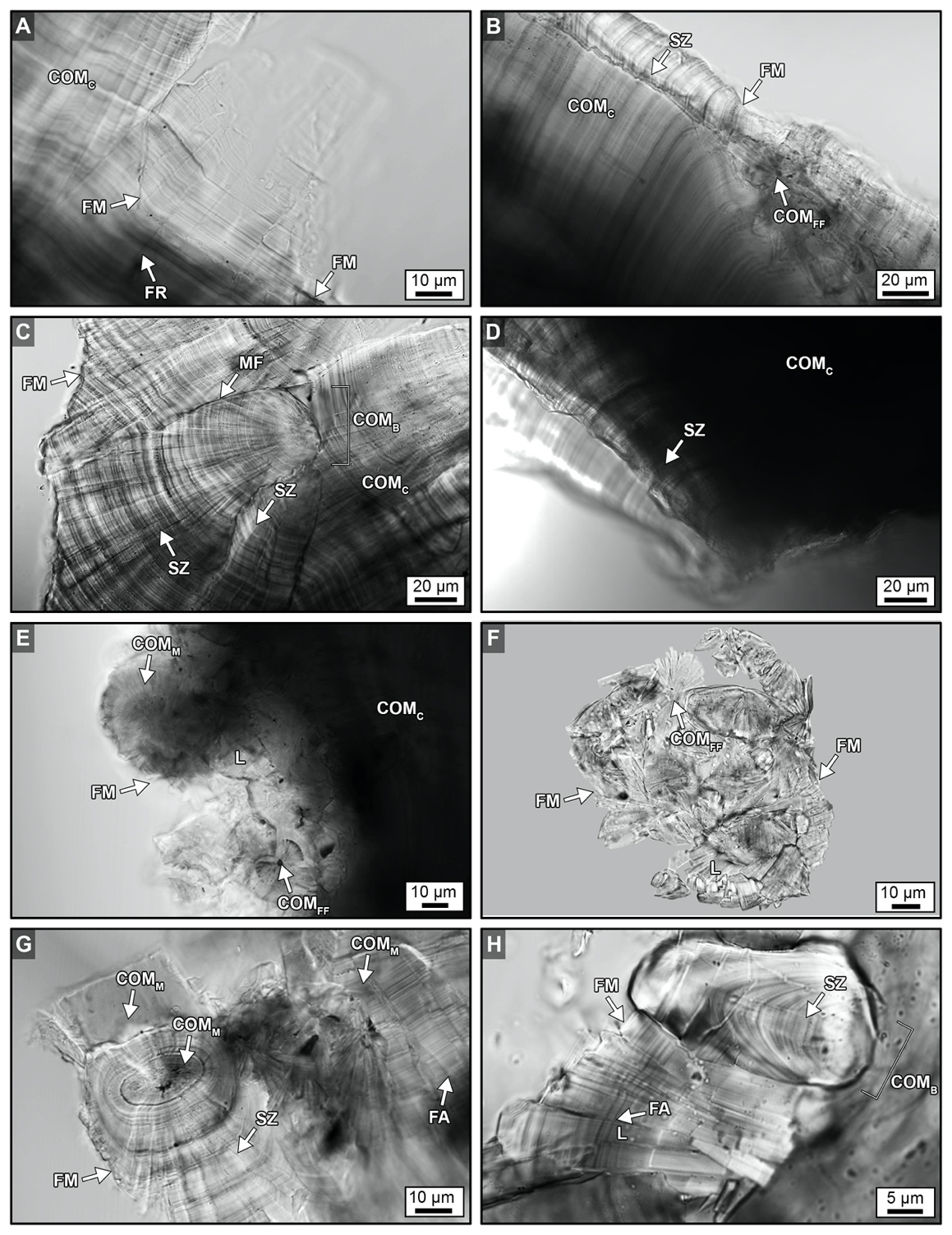


**Supplementary Figure 8.** Crystalline architecture and fracture patterns of SWL-derived particles from groups 106F2-S1 and 106F4-S1 embedded in the epoxy plug. (**A**-**H**) T-PMT images. Labels indicate: FR, fracture; FM, fracture margin; FA, fault; L, lath; SZ, sector zone; COM_FF_, free-floating COM; COM_C_, COM cortex; COM_B_, bundles of COM radiating from a COM_FF_; MF, microfractures; COM_M_, mimetic replacement COM. Image locations shown in Figure 4C, F and corresponding CAF and SRAF figures presented in Figure 6.


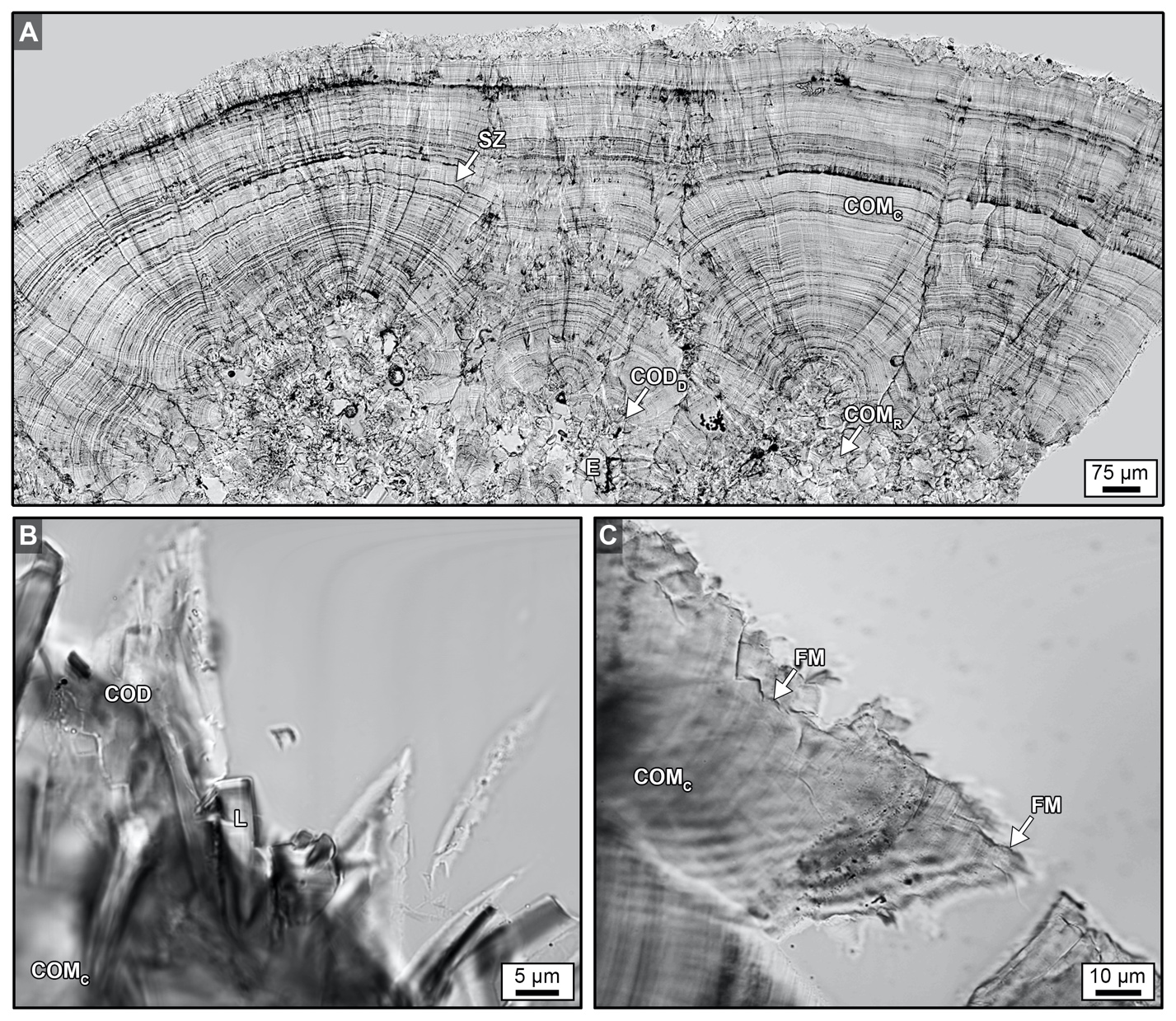


**Supplementary Figure 9.** T-PMT images (**A**-**H**) CaOx kidney stone crystalline architecture and fracture patterns. (A) MP2 CaOx kidney stone from Sivaguru et al. (2018). (**B**) Original growth margin containing COD and COM lathes. (**C**) Fracture margins of SWL-derived particles. Labels indicate: FM, fracture margin; L, lath; SZ, sector zone; COM_C_, COM cortex; COM_M_, mimetic replacement COM; COD; and COD_D_, dissolved COD. Image locations shown in Figure 4C, F and corresponding CAF and SRAF figures presented in Supplementary Figure 10.


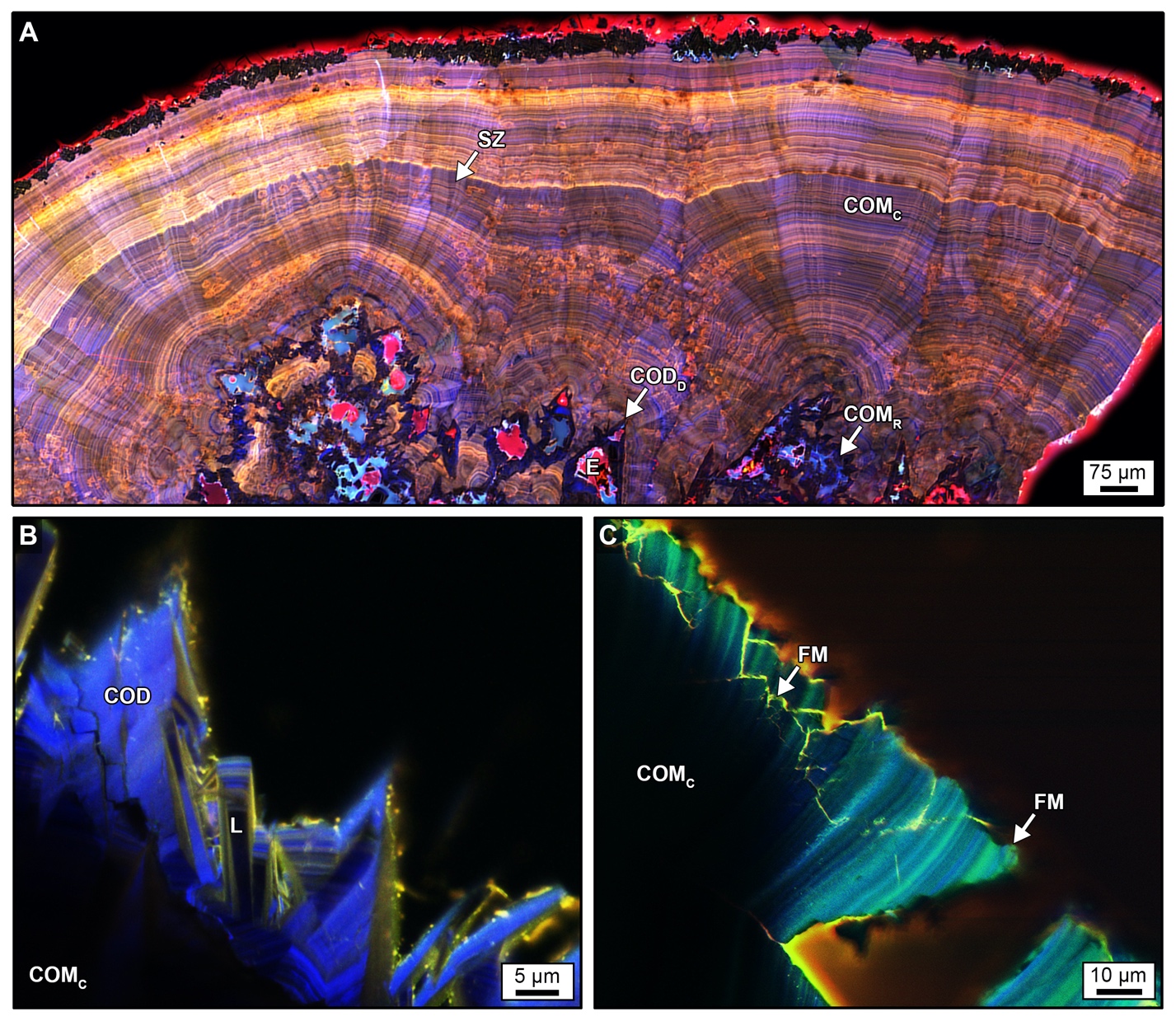


**Supplementary Figure 10.** CAF images (**A**, **C**) and SRAF (**B**) images of CaOx kidney stone crystalline architecture and fracture patterns. (A) MP2 CaOx kidney stone from Sivaguru et al. (2018). (**B**) Original growth margin containing COD and COM lathes. (**C**) Fracture margins of SWL-derived particles. Labels indicate: FM, fracture margin; L, lath; SZ, sector zone; COM_C_, COM cortex; COM_M_, mimetic replacement COM; COD; and COD_D_, dissolved COD. Corresponding T-PMT figures presented in Supplementary Figure 9.

1. **Supplementary Tables**


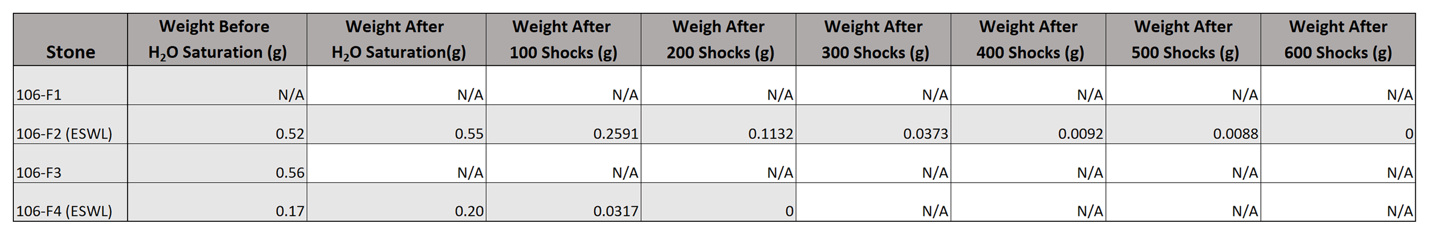


**Supplementary Table 1.** Weight of PCNL-derived fragments (106F3, 106F2, and 106F4), change in weight of PCNL-derived fragments 106F2 and 106F4 after 72-hours of H_2_O saturation prior to SWL experimentation, and weight changes during experimentation in SWL-derived particles during each incremental 100-shock treatment. Complementary RL images and data are presented in Figures 2A, 4, 7A and Supplementary Figures 1A, 2 and 4.


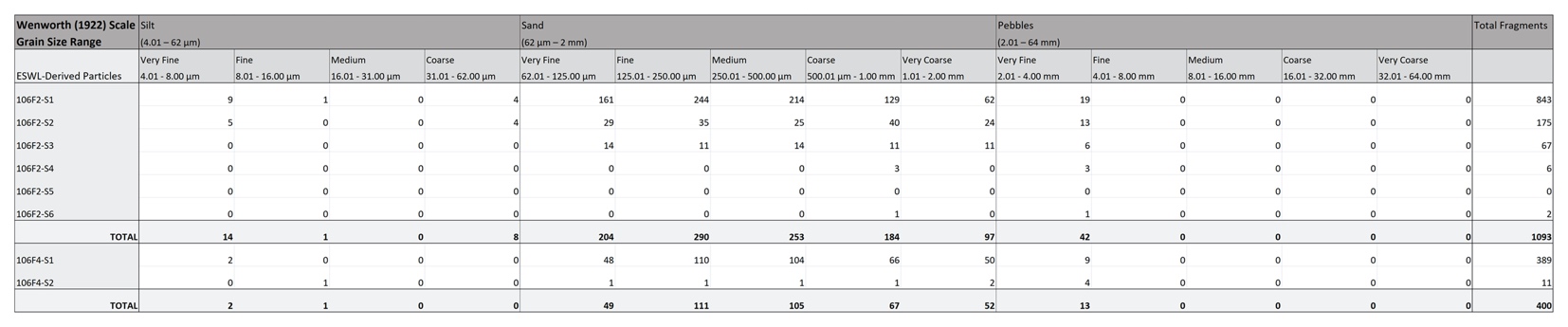


­­

**Supplementary Table 2.** Wentworth grain size frequency distribution of SWL-derived particles from 106F2 and 106F4 after incremental SWL 100-shock treatments. Complementary data presented in Figure 7B, C.

| Grain Size (cm) | 1 | 0.2 | 0.0064 | 0.0004 | 0.000014 |
| --- | --- | --- | --- | --- | --- |
| Grain Size (μm) | 10000 | 2000 | 64 | 4 | 0.14 |
| Surface Area (m^2^/g) | 0.000283 | 0.0014151 | 0.0442217 | 0.7075472 | 20.215633 |

**Supplementary Table 3.** Wentworth Grain Size Surface Area Calculations. Complementary data to Figure 8.

| 24-Hour Urine Collection Date | Osmolality  (mOsm/kg) | pH | SS,  CaOx  (DG) | SS,  Brushite  (DG) | SS,  Hydroxyapatite  (DG) | SS,  Uric Acid  (DG) | SS,  Sodium Urate  (DG) |
| --- | --- | --- | --- | --- | --- | --- | --- |
| 1/16/2012 | 461 | 6.2 | 1.66 | -0.60 | 3.88 | -1.54 | 1.39 |
| 4/11/2012 | 313 | 5.7 | 1.31 | -0.96 | 2.25 | **↑** 1.70 | 0.21 |
| 6/24/2013 | 364 | 5.5 | **↑** 1.92 | -2.44 | 0.43 | **↑** 2.25 | -0.04 |
| 6/30/2014 | 344 | 5.1 | **↑** 2.08 | -3.67 | -1.10 | **↑** 2.30 | -0.71 |

**Supplementary Table 4.** 24-Hour Urine Supersaturation Profile for Patient 106. Acronyms includes SS, supersaturation; and DG, Delta G energy. Arrows indicate above normal concentration range.

| 24-Hour Urine Collection Date | Na^+^  (mmol/  24 hrs) | K^+^  (mmol/  24 hrs) | Ca^+^  (mg/  24 hrs) | Mg^+^  (mg/  24 hrs) | Cl^-^  (mmol/  24 hrs) | PO_4_^3-^  (mg/  24 hrs) | SO_4_^2-^  (mmol/  24 hrs) | Citrate  (mg/ 24 hrs) | Oxalate  (mmol/  24 hrs) | Cr  (mg/  24 hrs) |
| --- | --- | --- | --- | --- | --- | --- | --- | --- | --- | --- |
| 1/16/2012 | **↑** 423 | 68 | 201 | 115 | **↑** 441 | 833 | 12 | **↓** 336 | **↑** 0.59 | 1173 |
| 4/11/2012 | -136 | 43 | 173 | 143 | 113 | **↑** 1503 | 16 | **↓** 363 | 0.37 | 1247 |
| 6/24/2013 | 132 | **↑** 87 | 102 | 94 | 127 | **↑** 1121 | 20 | 354 | **↑** 0.84 | 1248 |
| 6/30/2014 | 224 | 56 | 172 | 101 | **↑** 241 | 677 | 20 | 91 | **↑** 0.71 | 883 |

**Supplementary Table 5.** 24-Hour Urine Supersaturation Profile for Patient 106. Abbreviations include: Cr, creatine. Arrows indicate above or below normal concentration range.
